# Zinc-solubilizing *Bacillus* spp. in conjunction with chemical fertilizers enhance growth, yield, nutrient content, and zinc biofortification in wheat crop

**DOI:** 10.3389/fmicb.2023.1210938

**Published:** 2023-07-04

**Authors:** Ramesh Chandra Yadav, Sushil K. Sharma, Ajit Varma, Udai B. Singh, Adarsh Kumar, Ingudam Bhupenchandra, Jai P. Rai, Pawan K. Sharma, Harsh V. Singh

**Affiliations:** ^1^Amity Institute of Microbial Technology, Amity University, Noida, Uttar Pradesh, India; ^2^Plant-Microbe Interaction and Rhizosphere Biology Lab, ICAR-National Bureau of Agriculturally Important Microorganisms, Kushmaur, Uttar Pradesh, India; ^3^Farm Science Centre, ICAR-Research Complex for North Eastern Hill Region, Tamenglong, Manipur, India; ^4^Department of Mycology and Plant Pathology, Institute of Agricultural Sciences, Banaras Hindu University, Varanasi, Uttar Pradesh, India

**Keywords:** microbial inoculants, nutrient use efficiency, wheat (*Triticum aestivum*), Zn biofortification, PGPR, rhizosphere, biological yield

## Abstract

Micronutrient deficiency is a serious health issue in resource-poor human populations worldwide, which is responsible for the death of millions of women and underage children in most developing countries. Zinc (Zn) malnutrition in middle- and lower-class families is rampant when daily calorie intake of staple cereals contains extremely low concentrations of micronutrients, especially Zn and Fe. Looking at the importance of the problem, the present investigation aimed to enhance the growth, yield, nutrient status, and biofortification of wheat crop by inoculation of native zinc-solubilizing *Bacillus* spp. in conjunction with soil-applied fertilizers (NPK) and zinc phosphate in saline soil. In this study, 175 bacterial isolates were recovered from the rhizosphere of wheat grown in the eastern parts of the Indo-Gangetic Plain of India. These isolates were further screened for Zn solubilization potential using sparingly insoluble zinc carbonate (ZnCO_3_), zinc oxide (ZnO), and zinc phosphate {Zn_3_(PO_4_)_2_} as a source of Zn under *in vitro* conditions. Of 175 bacterial isolates, 42 were found to solubilize either one or two or all the three insoluble Zn compounds, and subsequently, these isolates were identified based on 16S rRNA gene sequences. Based on zone halo diameter, solubilization efficiency, and amount of solubilized zinc, six potential bacterial strains, i.e., *Bacillus altitudinis* AJW-3, *B. subtilis* ABW-30, *B. megaterium* CHW-22, *B. licheniformis* MJW-38, *Brevibacillus borstelensis* CHW-2, and *B. xiamenensis* BLW-7, were further shortlisted for pot- and field-level evaluation in wheat crop. The results of the present investigation clearly indicated that these inoculants not only increase plant growth but also enhance the yield and yield attributes. Furthermore, bacterial inoculation also enhanced available nutrients and microbial activity in the wheat rhizosphere under pot experiments. It was observed that the application of *B. megaterium* CHW-22 significantly increased the Zn content in wheat straw and grains along with other nutrients (N, P, K, Fe, Cu, and Mn) followed by *B. licheniformis* MJW-38 as compared to other inoculants. By and large, similar observations were recorded under field conditions. Interestingly, when comparing the nutrient use efficiency (NUE) of wheat, bacterial inoculants showed their potential in enhancing the NUE in a greater way, which was further confirmed by correlation and principal component analyses. This study apparently provides evidence of Zn biofortification in wheat upon bacterial inoculation in conjunction with chemical fertilizers and zinc phosphate in degraded soil under both nethouse and field conditions.

## Introduction

Globally, wheat (*Triticum aestivum* L.) is the most important staple crop, and it is considered a main source of food and income for millions of smallholder farmers (Shewry and Hey, 2015). It is grown in nearly every part of the world. Wheat-based foods are therefore critical for food and nutritional security worldwide (FAOStat, http://faostat.fao.org/site/291/default.aspx). In addition, wheat is considered an important staple food as well as a major source of food, feed, fiber, fuel, starch, and energy (Paloma et al., 2016; Acevedo et al., 2018; Singh et al., 2019; Igrejas and Branlard, 2020). It also provides substantial amounts of other nutritional components, which are essential or beneficial for health. Among them, vitamins (notably the B group of vitamins), dietary protein, fiber, minerals, and phytochemicals are noteworthy (Igrejas and Branlard, 2020; Erenstein et al., 2022). There is a well-established relationship between consumption of cereal dietary fiber and reduced risk of cardiovascular disease, type 2 diabetes, and forms of cancer (notably colorectal cancer) (Pipero et al., 2015). Besides these, zinc (Zn) deficiency in wheat is rampant and causes Zn malnutrition in resource-poor human populations especially in women and underage children in most developing countries (Mottaleb et al., 2022). It has been reported that one-third (3 billion) of the global population is at high risk of Zn deficiency (FAO, 2014). Such an inadequate dietary status of Zn induces diseases such as immune deficiency, cancer, memory disorder, pneumonia, cardiovascular disorder, respiratory issues, and diarrhea in humans (Yadav et al., 2022). Therefore, mitigating Zn malnutrition poses a big challenge for researchers, policymakers, and other stakeholders. Zinc is a vital micronutrient for plants regulating the production of phytohormones, synthesis of starch, chlorophyll, protein, maintenance of membrane integrity, carbohydrate metabolism, auxin metabolism, cell growth, and multiplication (Alloway, 2008). Furthermore, it is also a cofactor of all six classes of enzymes, namely hydrolases, isomerases, ligases, lyases, oxidoreductases, and transferases (Rehman et al., 2019; Ullah et al., 2020). Normal plant growth and development require around 15–55 μg g^−1^ of zinc in the tissues (Al Jabri, 2022). Zn deficiency in plants induces chlorosis, retards growth, affects root development, water uptake and transport, and grain yield, and affects the immune responses against biotic and abiotic responses in plants (Alloway, 2004). The application of an optimal dose of Zn improves the quality of cereal grains by improving proteins, carbohydrates, nucleic acid, and lipid synthesis (Sharma et al., 2013).

There are several factors, such as agronomic, edaphic, environmental, and anthropogenic, responsible for zinc deficiency in 50% of the world's soils (Alloway, 2009; Ramesh et al., 2014). Similarly, there are soil factors responsible for the low availability of Zn to plants, such as low organic matter contents, high calcium carbonate content, extremely high and low pH, significantly higher concentrations of cations, and bicarbonate and phosphate concentration being the noteworthy ones (Alloway, 2009; Rehman et al., 2018a,b; Singh et al., 2021b). Increasing the uptake of Zn from soil and accumulation in the grain can reduce the problem of Zn deficiency. Grain enrichment with Zn can be enhanced through agricultural approaches such as agronomic, plant breeding, and transgenic approaches (Prasad et al., 2010). Of these, continuous application of Zn fertilizers in the soil leads to its fixation into unavailable form in soil subject to the soil types and chemical reactions (Cakmak, 2008). Such insoluble/unavailable Zn compounds in the soil can be transformed back to soluble/available form by the processes governed by zinc-solubilizing bacteria (ZSB) (Saravanan et al., 2007; Aloo et al., 2022; Yadav et al., 2022). The use of ZSB is an environmentally friendly, cost-effective, and sustainable approach for Zn biofortification in cereal grain crops. Several ZSB strains have been reported to solubilize unavailable forms of Zn for improving plant growth, yield, and grain quality (Ramesh et al., 2014; Yadav R. et al., 2020). Among these, *Bacillus* spp., ubiquitous in nature, are widely studied that possess a multitude of PGP traits, including zinc solubilization. *Bacillus* spp. solubilize insoluble forms of Zn compounds by the secretion of organic acids in general, proton extrusion, and production of chelating ligands (Zhao et al., 2011; Rashid et al., 2012; Ramesh et al., 2014; Yadav et al., 2022). Production of 2-ketogluconic acid and gluconic acid by ZSB is of particular mention as these are the main acids responsible for Zn solubilization (Zhao et al., 2011; Desai et al., 2012; Rashid et al., 2012; Kumari et al., 2016; Yadav et al., 2022). According to earlier studies, inoculation of wheat with *Bacillus aryabhattai* MDSR14 and *B. thuringiensis* FA-4 enriched grain's Zn up to 38 and 46%, respectively (Ramesh et al., 2014; Abaid-Ullah et al., 2015).

Biofortification by inoculation of ZSB is a widely accepted approach for enriching nutrient concentration in the edible portions of the crops to improve human and animal health (Aloo et al., 2022). In view of the abovementioned facts, it is imperative to evaluate the effectiveness and efficacy of ZSB in a sustainable way to increase the bioavailability of Zn in soil, which ultimately helps in maintaining plant and human health (Igrejas et al., 2020). The information on the effects of rhizobacteria especially bacilli in conjunction with the recommended dose of fertilizer (RDF) and zinc phosphate on Zn biofortification in wheat is lacking, and this study was undertaken in order to develop an agronomic practice for wheat cultivation in degraded soil of Eastern Uttar Pradesh and also other parts of the country. Hence, the objectives of the study were to identify and characterize bacteria isolated from wheat rhizosphere with zinc-solubilizing ability and to further evaluate the influence of single inoculation of potential zinc-solubilizing rhizobacterial in conjunction with soil-applied RDF and Zn phosphate in degraded soil on growth, yield, and Zn biofortification of wheat crop under both pot and field conditions.

## Materials and methods

### Bacterial strains

During the course of the investigation, 32 soil samples from the rhizosphere of wheat were collected from different parts of Eastern Uttar Pradesh, India into cool packs and brought to the laboratory. The moist soil samples were stored in a refrigerator until further analysis. For the isolation of bacterial isolates, serially diluted soil samples were plated on nutrient agar, Bacillus agar, and Pseudomonas agar (HiMedia Pvt. Ltd., Mumbai, India) with incubation at 28°C for 2-3 days. One hundred seventy-five isolates with diverse morphotypes were recovered from 32 soil samples. The pure bacterial cultures were maintained on a nutrient agar medium at 28°C till further use. Later on, another duplicate set of cultures was preserved in 20% glycerol stock at−20°C in a deep freezer (Blue Star, India).

### *In vitro* screening of zinc-solubilizing ability of rhizobacteria

These rhizobacterial isolates were screened for their zinc-solubilizing ability on tris-minimal agar medium with D-glucose (10 gl^−1^) and separately supplemented with zinc oxide (1.244 g l^−1^ = 15.23 mM), zinc phosphate (1.9882 g l^−1^ = 5.0 mM), and zinc carbonate (1.728 g l^−1^ = 5.2 mM) to prepare three separate media (Khande et al., 2017) with slight modifications (Yadav et al., 2022). Briefly, freshly grown bacteria were spot-inoculated with doubled sterile toothpicks on Petri plates containing tris-minimal agar medium separately amended with zinc oxide, zinc phosphate, and zinc carbonate. The Petri plates were incubated in the dark at 28°C for 7 days to observe the formation of a clear halo zone around colonies. Subsequently, the colony diameter and the diameter of the halo zone (mm) formed around the colony were measured after 7 days of inoculation.

### Identification of zinc-solubilizing rhizobacteria

Based on the zinc solubilization potential of the isolates, 42 isolates were shortlisted and subjected to identification on the basis of 16S rRNA gene sequence homology. Bacterial DNA was isolated using Nocleo-pore^®^ gDNA Fungal/Bacterial Mini Kit (Cat.# NP-7006D, Genetix Biotech Asia Pvt. Ltd., New Delhi, India). According to Singh et al. (2021a), the amplification of 16S rRNA gene was performed using Peqlab peqSTAR, Thermal Cyclers (VWR Lab Products Pvt. Ltd., Bangalore, India). The molecular grade chemicals used were procured from Merck Specialities Private Limited, Mumbai, India. The sequences were aligned using the EzTaxon server for the identification of the bacterial isolates. The phylogenetic analysis of sequences was performed using Molecular Evolutionary Genetics Analysis (MEGA- 10), and 16S rRNA gene sequences were submitted to the NCBI GenBank.

### Selection of potential Zn-solubilizing rhizobacteria

The isolates which solubilized all three zinc compounds were considered potential zinc solubilizers (Yadav et al., 2022). Based on the zone diameter formed on all three solid media, six potential zinc solubilizers were further evaluated for their ability to release soluble zinc in a liquid medium as well as a reduction in medium pH in Erlenmeyer flasks (Ramesh et al., 2014; Khande et al., 2017). Briefly, 1.0 ml culture (10^8^ CFU ml^−1^) of each bacterium was inoculated in tris-minimal broth containing 0.1% Zn as zinc oxide, zinc phosphate, and zinc carbonate, separately. The tris-minimal broth supplemented with inorganic zinc but without bacterial inoculation served as an uninoculated control. After 10 days of incubation at 28°C in a shaker at 120 rpm, the samples were withdrawn and centrifuged at 10,000 rpm for 10–12 min to obtain clear supernatant, which was directly fed to an atomic absorption spectrophotometer for estimation concentration of soluble zinc (μg Zn ml^−1^). The fall in pH of the broth medium of all the treatments including uninoculated was also measured. Entire experiments were performed in triplicates. Furthermore, Zn solubilization efficacy was calculated according to Vazquez et al. (2000) with slight modification (Ramesh et al., 2014). The gluconic acid was quantified using high-performance liquid chromatography (Shimadzu, Separon SGX C18 column) equipped with a quaternary pump, auto-sampler, DAD detector, and degasser following the procedure described by Larcher et al. (2009) with slight modifications (Sunithakumari et al., 2016).

These strains were further evaluated for their plant growth-promoting attributes, such as solubilization of potassium and phosphate; and production of indole-3-acetic acid (IAA), siderophore, ammonia, and HCN of potential zinc solubilizers were assayed using standard methods. The potassium solubilization was estimated by the method of Rajawat et al. (2016), whereas phosphate solubilization was performed on Pikovskaya medium containing 0.1% tri-calcium phosphate according to Olsen and Sommers (as cited in Penrose and Glick, 2003). The IAA production by zinc solubilizers was estimated as per the method of Ahmad et al. (2020). The siderophore production on chrome azurol S (CAS) agar by zinc solubilizers was estimated as per the method described by Schwyn and Neilands (1987). HCN production was estimated as per the method of Kremer and Souissi (2001). Ammonia production of zinc solubilizers was estimated by adding 1 ml Nessler's reagent to 72-h-old cultures grown in peptone water broth. Production of the brown color from yellow was treated as a positive test. ACC deaminase activity of zinc solubilizers was determined as per the method of Bal et al. (2013).

The catalase, oxidase, amylase, protease, and lipase enzyme potential of zinc solubilizers was tested using standard methods (Cappuccino and Welsh, 2017). Cellulase production was tested by growing bacterial cultures on LB agar supplemented with 1% carboxymethyl cellulose (CMC) using the method described by Lin et al. (2012), whereas lipase was detected on LB agar supplemented with Tween-20 (Sierra, 1957). Skimmed milk agar was used for the production of proteases (Kumar et al., 2005).

### Evaluation of potential zinc solubilizers under pot and field conditions

#### Planting materials and growth conditions

Wheat seeds (cv. HD-2967) were procured from ICAR-Indian Institute of Seed Sciences, Kushmaur, Mau, India. Experiments were conducted during the winter season (2020–2021 and 2021–2022) to investigate the impact of zinc-solubilizing bacteria on wheat growth and development and Zn biofortification. The weather conditions during the growing period were as follows: mean temperature of 22-25°C and relative humidity of 70–75% with 11-/13-h photoperiod.

#### Preparation of inoculants of zinc solubilizers

Six selected zinc-solubilizing bacterial strains, *viz*., *B. altitudinis* AJW-3, *B. subtilis* ABW-30, *B. megaterium* CHW-22, *B. licheniformis* MJW-38, *Brevibacillus borstelensis* CHW-2, and *B. xiamenensis* BLW-7, were used for their evaluation on plant growth, yield, biofortification of nutrient content, and rhizosphere properties of wheat crop. The bacterial inoculums were prepared using 0.85% sterile saline solution as per the method described by Ramesh et al. (2014) with slight modifications (Singh et al., 2016). The population count (CFU) of inoculums was adjusted to 2 × 10^8^ cfu ml^−1^ before application to the seeds.

#### Experimental setup

The six selected zinc-solubilizing bacterial strains were evaluated in the presence of RDF and with or without zinc phosphate under the nethouse at ICAR-NBAIM, Mau (25°53”56.99′′N 83°29”18.29′′E; elevation 74 m) as well as under farmers' field conditions near District Jail, Mau (25°55.828 N 83°28.390 E; elevation: 52m), Uttar Pradesh, India, to assess their impact on plant growth, yield, and biofortification of nutrient content especially Zn in wheat grains. The eight treatments were: T_1_–Absolute control, T_2_–RDF, T_3_–RDF + *B. altitudinis* AJW-3, T_4_–RDF + *B. subtilis* ABW-30, T_5_–RDF + *B. megaterium* CHW-22, and T_6_–RDF + *B. licheniformis* MJW-38, T_7_–RDF + *Brevibacillus borstelensis* CHW-2, and T_8_–RDF + *B. xiamenensis* BLW-7. The experiment was performed in a completely randomized block design (CRBD)/randomized block design (RBD) containing eight different treatments with five replicates each in the nethouse and three replicates each in the field experiment. Recommended doses of fertilizers for N: P: K (120:60:60) were applied in all treatments. The experiment was divided into two parts, the first part with the above eight treatments where the wheat (variety HD-2967) seeds were treated with zinc-solubilizing bacterial strains in soil without zinc phosphate [Zn_3_ (PO_4_)_2_]. Another part of the experiment included similar treatments but the soil was applied with 5 kg/ha zinc phosphate to study the effect of combined inoculation of soil-applied zinc and inoculation of zinc-solubilizing bacteria on plant growth yield and nutrient content in soil and plant. Moisture content in the pots was maintained at field capacity by adding sterile distilled water as and when needed.

### Evaluation of zinc-solubilizing *Bacillus* spp. in pots under nethouse conditions

The experiment was laid out with eight treatments and five replications each in a complete randomized design block (CRBD) with or without zinc phosphate application. The treatments are the same as mentioned in the previous section, “Experimental setup.” Soil for the pot experiments was collected from farmers' fields near the District Jail, Mau (25°55.828 N 83°28.390 E, Elevation: 52m), Uttar Pradesh, India, and each pot was filled up with 3 kg soil. The characteristics of the experimental soil were as follows: pH 8.2 ± 0.2, EC 4.2 ± 0.2 dS m^−1^, OC 0.37 %, available-N 142.50 kg ha^−1^, available-P 13.96 kg ha^−1^, available-K 205.56 kg ha^−1^, DTPA-Zn 0.52 μg g^−1^. Recommended doses of fertilizers for N: P: K (120:60:60) kg/ha were applied as basal doses. The soil was further mixed with 6.6 mg Zn_3_ (PO_4_)_2_ (at the rate of 2.2 mg Kg^−1^) as a Zn source in each pot containing 3 kg soil in one set of experiments. Furthermore, seeds were surface-sterilized with 1% NaOCl for 60 s and later washed with sterile distilled water 3-4 times to make seeds free of NaOCl. A population of 10^8^ CFU ml^−1^ bacterial suspension was prepared using 0.85% sterile saline solution as mentioned earlier.

Seeds were bioprimed with 20 ml of bacterial suspension/kg^−1^ seeds for overnight (12 h) at ambient room temperature followed by the sowing of four seeds per pot. After germination, only two seeds were maintained during the entire experimentation following all standard agronomic practices. All the pots were irrigated as per the requirement with sterile distilled water to maintain moisture at field capacity.

### Evaluation of zinc solubilizers in the field

A field experiment with wheat was conducted in the plot (dimension 3 × 2 m^2^) with plot-to-plot spacing of 1 m during the winter season (2020–2021 and 2021–2022). The randomization of plots with three replications was maintained. Each plot was basely dressed with N: P: K (120:60:60 kg ha^−1^), and zinc phosphate was added as described in the previous section. The field soil was deep and well-drained silty clay loam with pH 8.2 ± 0.2, EC 4.2 ± 0.2 dS m^−1^, OC 0.37 %, available-N 142.50 kg ha^−1^, available-P 13.96 kg ha^−1^, available-K 205.56 kg ha^−1^, and DTPA-Zn 0.52 μg g^−1^. The same wheat variety HD-2967 that was grown in the pot experiment was used in the field study. Seeds were sown at a rate of 43 seeds m^−2^ and a depth of 4–5 cm. The treatments were the same as mentioned in the section on “Experimental setup”. Bacterially treated seed @ 120 kg ha^−1^ was sown to each plot in line. All the standard agronomic practices prescribed for wheat crop were followed. Four irrigations were given at critical stages, i.e., crown root initiation stage (21 DAS), tillering stage (45 DAS), flowering stage (70 DAS), and grain filling stage (95 DAS) to fulfill the water requirement of the crop under field conditions.

### Evaluation and analysis

#### Effects of seed biopriming on plant growth and yield parameters

The wheat plants were uprooted from both pot and field carefully, and data were recorded on the agronomic parameters such as plant height (cm) and dry matter accumulation (g pot^−1^ and g m^−2^ under field conditions) at 30DAS, 60DAS, and 90DAS (days of sowing) as the indicative of plant growth according to Kumar et al. (2014), and yield attributes such as number of effective tillers, spike lengths (cm), spikelet spike^−1^, number of grain spike^−1^, test weight, and grain yield of wheat were measured after harvesting as per the methods described by Rana et al. (2014). The grains were dried in sunlight for 4–5 days, carefully weighed, and the yield was calculated in terms of tones hectare^−1^. The grain yield was noted at 10% moisture content. The straw yield and biological yield were measured in tons per hectare, and the Harvesting Index (HI) was calculated in percentage as per the method given by Kumar et al. (2014).

#### Effects of seed biopriming on chemical and biological properties of soil

Soil samples were collected after harvesting wheat plants under both pot and field conditions and subjected to analysis of soil chemical and biological properties. Before analysis, all the soil samples were air-dried and passed through a 1-mm sieve. The soil's chemical properties such as soil pH (1:2.5 soil: water) were measured using a glass electrode pH meter (Richards, 1954), and the EC (dS m^−1^) of soil was measured using a conductivity bridge (Piper, 1950). The SOC was determined as per the method described by Walkley and Black (1934). Soil-available macronutrients N, P, and K were determined by the standard method described by Subbiah and Asija (1956), Olsen (1954), and Jackson (1973), respectively. The micronutrients, *viz*., Zn, Fe, Mn, and Cu concentrations in soil were determined by the DTPA extraction method using an atomic absorption spectrophotometer (Lindsay and Norvell, 1978). The soil biological properties such as soil microbial biomass (SMBC mg kg–^1^ soil), soil dehydrogenase activity (DHA, μg TPF g^−1^ soil day–^1^), soil alkaline phosphatase activity (APA, μg *p*-nitrophenol g^−1^ soil h^−1^), and fluorescein diacetate (FDA, μg fluorescein released g^−1^ soil h^−1^) were measured following the standard method described by Casida et al. (1964), Tabatabai and Bremner (1969), Nunan et al. (1998), and Green et al. (2006), respectively.

#### Effects of seed biopriming on macro- and micronutrients in plant samples

At maturity, the wheat plants under pot and field experiments were sampled for the estimation of macro- (N, P, and K) and micronutrient (Zn, Fe, Cu, and Mn) contents. The plant samples (grains and straw) were dried, ground to fine powder, and digested in a di-acid mixture containing nitric acid and perchloric acid (5:4 v/v) at 320°C for 1 h, and Zn, Fe, Cu, and Mn content (μg Zn g^−1^ plant material) was measured using an atomic absorption spectrophotometer at the most sensitive wavelengths for Zn (213.7 nm), Fe (248.7 nm), Cu (324.6 nm), and Mn (279.5 nm). Potassium concentration was analyzed using a flame photometer and compared with standards ranging from 0 to 100 ppm of KCl. Phosphorous concentration was measured using the method of Jackson (1967). The total N of plant samples was estimated using the Kjeldahl method (Jackson, 1967).

#### Effects of seed biopriming on nutrient use efficiency of wheat plant

The nutrient use efficiency such as partial factor productivity, agronomic efficiency, apart nutrient recovery, and physiological efficiency of wheat plants was calculated as per the method described by Piper (1966).

### Statistical analysis

The nethouse and field experiments were repeated twice in two consecutive years (2020–2021 and 2021–2022), and pooled analysis was performed. The data on various parameters were analyzed in triplicates for the plant study and subjected to an analysis of variance (ANOVA) in accordance with the experimental design (completely randomized block design) using Statistical Package for Social Science (SPSS) version 11.5 to quantify and evaluate the source of variation. The treatment means were compared at a significance level of 0.05. Furthermore, all the data obtained from this study were statistically analyzed using the F-test as per the procedure described by Gomez and Gomez (1984). Least significance difference (LSD) values at *p*=0.05 were used to determine the significance of differences between means. Correlation analysis was performed using SPSS 11.5. Heatmaps were constructed using Package ‘Heatplus' version 3.7.0 (Ploner, 2020), ‘RColorBrewer' (Kanno et al., 2011), and ‘g plots' (Warnes et al., 2016) packages in R (version 3.6.1) using Ward's hierarchical clustering (Strauss and von Maltitz, 2017). The PCA was conducted using the biplot method in Matlab R2019b version 9.7 (Math Works Inc., USA) to detect the effect of different treatments on the Zn and other nutrient concentrations (N, P, K, Fe, Cu, and Mn) in grains and straw of wheat crops. The PCA was carried out according to Mishra et al. (2017). PCs with high eigenvalues best signify variation in the systems, so only the PCs with eigenvalues ≥ 1 were retained (Kaiser, 1960). The extracted outcomes of a PCA are presented in terms of component scores, also known as factor scores and loadings (Wold et al., 1987).

## Results

### Isolation and identification of zinc-solubilizing rhizobacteria

**Figure 1:**
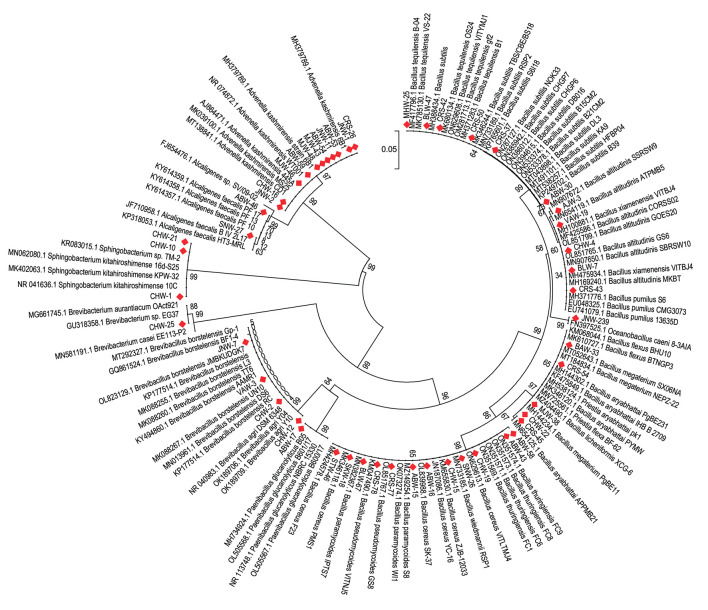
Neighbor joining tree derived using CLUSTAL W and MEGA X using analysis of 16S rRNA gene sequences. The numbers at nodes indicate bootstrap support values, as calculated using MEGA 5.2.

During the course of isolation, 175 distinct bacterial morphotypes were isolated from different parts of Eastern Uttar Pradesh. These isolates were screened for Zn solubilization on tris-minimal agar medium supplemented with three different Zn sources, i.e., zinc oxide, zinc carbonate, and zinc phosphate. Of the 175 morphotypes, 42 rhizobacteria solubilized either of the zinc compounds on plate assay and were shortlisted for identification (Supplementary Table 1). The isolates were designated as CHW-12, ABW-30, CHW-21, JNW-2, VAW-19, ABW-43, ABW-46, CHW-4, JNW-23, BLW-7, ABW-17, SNW-27, CHW-16, CHW-2, MJW-46, ABW-59, CHW-19, MJW-48, MJW-43, ABW-54, CHW-25, SNW-26, ABW-58, CHW-15, CHW-10, CHW-22, JNW-11, ABW-16, ABW-53, ABW-15, JNW-1, VAW-3, MJW-38, JNW-7, AJW-3, CHW-1, BLW-47, BAW-33, BLW-67, SKW-18, MHW-25, and STW-46 (Supplementary Table 2). These 42 isolates were identified based on 16S rRNA gene sequence homology and percentage similarity during BLAST analysis using EzBioCloud, a public database of type strains (Supplementary Table 2). The 16S rRNA gene sequences were submitted to NCBI GenBank, and accession numbers were obtained. The results of BLAST homology analyses yielded 19 different bacterial species, including *Brevibacillus agri* (1), *Bacillus subtilis* (1), *Sphingobacterium kitahiroshimense* (3), *Advenella kashmirensis* (10), *Bacillus xiamenensis* (2), *Bacillus thuringiensis* (1), *Alcaligenes faecalis* (2), *Bacillus altitudinis* (2), *Oceanobacillus caeni* (1), *Paenibacillus glucanolyticus* (1), *Brevibacillus borstelensis* (3), *Bacillus cereus* (6), *Brevibacterium aurantiacum* (1), *Bacillus wiedmannii* (1), *Bacillus megaterium* or *Priestia megaterium* (1), *Bacillus paramycoides* (2), *Bacillus licheniformis* (1), *Bacillus tequilensis* (2) and *Bacillus flexus* or *Priestia flexa* (1). It was further observed that *Advenella kashmirensis* followed by *Bacillus cereus, Sphingobacterium kitahiroshimense* and *Brevibacillus borstelensis*, were the most dominant species (Figure 1, Supplementary Table 2).

### Characterization of zinc-solubilizing *Bacillus* spp.

It was observed that some of the strains solubilize all three zinc compounds tested. Based on the size of the halo zone formed around the bacterial colony and isolates solubilized by all three zinc compounds, six potential strains, *B. altitudinis* AJW-3, *B. subtilis* ABW-30, *B. megaterium* CHW-22, *B. licheniformis* MJW-38, *Brevibacillus borstelensis* CHW-2, and *B. xiamenensis* BLW-7, were selected for further in-depth investigation (Supplementary Table 3).

The zinc solubilization efficacy was calculated on the basis of the diameter of the halo zone formed on a growth medium supplemented with three different zinc compounds, *viz*., zinc oxide, zinc phosphate, and zinc carbonate. Among the six strains selected, *B. licheniformis* MJW-38 showed maximum zinc phosphate solubilization efficiency (411.76%) followed by *B. megaterium* CHW-22 (375.00%) and *B. altitudinis* AJW-3 (325.00%). However, the least zinc phosphate solubilization efficiency was reported in *Brevibacillus borstelensis* CHW-2 (273.68). Similarly, *B. megaterium* CHW-22 (375.00%) showed maximum zinc oxide followed by *B. subtilis* ABW-30 (244.44%) and *B. xiamenensis* BLW-7 (225.00%). However, in the case of zinc carbonate, maximum solubilization efficacy was recorded in *B. megaterium* CHW-22 (244.95%) followed by *Brevibacillus borstelensis* CHW-2 (240.00%), *B. subtilis* ABW-30 (216.22%), and *B. xiamenensis* BLW-7 (214.29%) (Table 1).

**Table 1:** Zinc solubilization halo zone on agar medium, solubilization efficiency, zinc solubilization, pH, and gluconic acid production in liquid cultures containing zinc as inorganic insoluble form as influenced by inoculation of zinc-solubilizing *Bacillus* species.

**Isolates**	**Dia. of zinc solubilization halo zone (mm)**	**Solubilization efficiency (%)**	**Amount of solubilized zinc (μg Zn ml^−1^)**	**pH of culture medium**	**Gluconic acid (μg mL^−1^)**
**Zinc phosphate**					
Uninoculated control	NDc	ND	ND	6.8 ± 0.17^a^	ND
*Bacillus altitudinis* AJW-3	26.00 ± 1.72^c^	325.00 ± 13.8^c^	30 ± 0.90^c^	4.9 ± 0.13^c^	110.64 ± 2.35^e^
*Bacillus subtilis* ABW-30	24.00 ± 1.39^d^	282.00 ± 14.5^d^	28 ± 0.73^c^	5.2 ± 0.27^b^	120.87 ± 4.42^d^
*Bacillus megaterium* CHW-2	30.00 ± 2.72^a^	375.00 ± 9.82^ab^	38 ± 1.23^a^	4.3 ± 0.08^d^	150.92 ± 1.52^bc^
*Bacillus licheniformis* MJW-22	28.00 ± 1.65^ab^	411.76 ± 23.3^a^	40 ± 1.17^a^	4.2 ± 0.11^d^	129.93 ± 3.81^c^
*Brevibacillus borstelensis* CHW-38	26.00 ± 1.10^c^	273.68 ± 12.5^d^	25 ± 1.12^d^	5.4 ± 0.16^b^	171.57 ± 7.45^a^
*Bacillus xiamenensis* BLW-7	24.00 ± 0.98^d^	282.35 ± 8.35^d^	36 ± 1.43^b^	4.7 ± 0.22^c^	149.89 ± 2.38^bc^
**Zinc oxide**					
Uninoculated control	ND	ND	ND	6.9 ± 0.37^a^	ND
*Bacillus altitudinis* AJW-3	15.00 ± 0.45^d^	166.67 ± 6.42^d^	18 ± 0.73^c^	5.3 ± 0.39^bc^	141.61 ± 2.53^c^
*Bacillus subtilis* ABW-30	22.00 ± 0.92^a^	244.44 ± 9.35^a^	28 ± 0.65^a^	4.2 ± 0.15^ef^	119.95 ± 4.22^d^
*Bacillus megaterium* CHW-2	20.00 ± 1.42^ab^	250.00 ± 11.3^a^	29 ± 0.98^a^	4.4 ± 0.13^e^	164.75 ± 3.85^a^
*Bacillus licheniformis* MJW-22	14.00 ± 0.39^d^	215.38 ± 14.2^c^	24 ± 0.47^ab^	4.9 ± 0.08^d^	161.02 ± 3.25^ab^
*Brevibacillus borstelensis* CHW-38	16.00 ± 0.67^cd^	160.00 ± 7.22^d^	15 ± 0.52^d^	5.6 ± 0.25^b^	119.50 ± 5.42^d^
*Bacillus xiamenensis* BLW-7	18.00 ± 0.98^c^	225.00 ± 13.5^ab^	25 ± 1.33^ab^	4.8 ± 0.11^d^	161.47 ± 7.13^ab^
**Zinc carbonate**					
Uninoculated control	ND	ND	ND	6.8 ± 0.29^a^	ND
*Bacillus altitudinis* AJW-3	18.00 ± 0.53^c^	180.00 ± 5.42^c^	15 ± 0.62^d^	5.6 ± 0.22^b^	140.67 ± 2.44^b^
*Bacillus subtilis* ABW-30	20.00 ± 0.38^b^	216.22 ± 6.32^b^	20 ± 0.51^c^	5.2 ± 0.17^d^	76.62 ± 5.85^e^
*Bacillus megaterium* CHW-2	22.00 ± 0.49^ab^	244.44 ± 13.5^a^	24 ± 0.63^a^	4.3 ± 0.19^e^	153.17 ± 3.27^a^
*Bacillus licheniformis* MJW-22	16.00 ± 0.23^cd^	177.78 ± 8.75^c^	14 ± 0.88^d^	5.5 ± 0.28^bc^	144.40 ± 4.23^ab^
*Brevibacillus borstelensis* CHW-38	24.00 ± 0.75^a^	240.00 ± 9.32^a^	22 ± 1.08^ab^	4.6 ± 0.13^ef^	101.78 ± 3.57^d^
*Bacillus xiamenensis* BLW-7	15.00 ± 0.63^d^	214.29 ± 15.7^b^	19 ± 0.62^c^	5.4 ± 0.35^bc^	132.09 ± 4.62^c^

Data are mean values ± SD of three replicates; means with different letters in the same column differ significantly at *p* < 0.05 according to Fisher's LSD. ND, means not detected. Data with different letters show significant difference in column data in randomized block design test at *p* < 0.05 under Duncan's multiple range test.

In general, the maximum reduction in the media pH was reported in the case of *B. megaterium* CHW-22 across the zinc compounds used. The quantitative assays indicated that all six isolates are efficient zinc solubilizers. The maximum amount of solubilized zinc was recorded in a liquid medium inoculated with *B. licheniformis* MJW-38 (40.00 μg Zn ml^−1^) and supplemented with zinc phosphate at the lowest mean pH value of 4.2 (Table 1). In the case of zinc oxide, the maximum amount of solubilized zinc was recorded in a liquid medium inoculated with *B. megaterium* CHW-22 (29.00 μg Zn ml^−1^) and *B. subtilis* ABW-30 (28.00 μg Zn ml^−1^) and at the lowest mean pH value of 4.4 and 4.2, respectively. A more or less similar observation was recorded in the case of zinc carbonate where maximum zinc solubilization was reported in the liquid medium inoculated with *B. megaterium* CHW-22 (24.00 μg Zn ml^−1^) as compared to other strains tested (Table 1).

The production of gluconic acid in the presence of zinc phosphate, zinc oxide, and zinc carbonate by the rhizobacterial strains used in the study was determined using HPLC. All the strains were found to produce gluconic acid in the medium supplemented with zinc phosphate, zinc oxide, and zinc carbonate. The HPLC results clearly revealed that a significantly higher amount of gluconic acid was produced by *B. borstelensis* CHW-2 (171.57 μg ml^−1^) followed by *B. megaterium* CHW-22 (150.92 μg ml^−1^) and *B. xiamenensis* BLW-7 (149.89 μg ml^−1^) in the presence of zinc phosphate. A similar trend was recorded in the case of zinc oxide and zinc carbonate (Table 1).

Selected strains were further characterized for PGP traits, viz. P solubilization, K solubilization, IAA production, ACC deaminase activity, siderophore, HCN, and ammonia production. Similarly, these strains were also tested for the production of enzymes such as catalase, oxidase, amylase, protease, lipase, and cellulose (Supplementary Table 4). All six strains solubilized P and K and exhibited strong PGP traits. These strains were found to produce varying levels of IAA in culture filtrate with the maximum being by *B. megaterium* CHW-22 (18.61 μg ml^−1^) followed by *B. xiamenensis* BLW-7 (12.70 μg ml^−1^) and *B. licheniformis* MJW-38 (10.16 μg ml^−1^). Except for *B. altitudinis* AJW-3, *B. subtilis* ABW-30, and *B. megaterium* CHW-22, the other three strains did not produce ACC deaminase in the medium. Differential activities were recorded for siderophore, HCN, and ammonia production. A more or less similar trend was recorded for enzyme assay except for oxidase. None of the bacterial strains was found positive for the oxidase test (Supplementary Table 4).

### Evaluation of microbial inoculants under nethouse conditions

#### Plant growth and yield attributes

The effect of rhizobacterial inoculation on plant height and dry matter accumulation was recorded at 30, 60, and 90 DAS in the wheat plants grown under nethouse conditions supplemented with and without zinc phosphate. In general, all the strains considerably increased the plant height and dry matter accumulation as compared to absolute control plants and RDF-treated plants. When compared among the microbial inoculants, significantly higher plant height and dry matter accumulation were recorded in the treatments inoculated with *B. megaterium* CHW-22 closely followed by *B. licheniformis* MJW-38 and *B. altitudinis* AJW-3 as compared to other inoculants at 30, 60 and 90 DAS. A more or less similar trend was recorded in the plants grown with and without zinc phosphate. However, these parameters were slightly higher in the plants amended with zinc phosphate (Table 2).

**Table 2:** Effect of zinc-solubilizing plant growth promoting rhizobacteria on growth, yield attributes, and yield of wheat under pot experiment.

**Treatments**	**Plant height (cm)**			**DMA (g pot** ^**−1**^ **)**			**Yield attributes**					**GY** **(g pot^−1^)**	**SY (g pot^−1^)**	**BY** **(g pot^−1^)**	**HI (%)**
	**30 DAS**	**60 DAS**	**90 DAS**	**30 DAS**	**60 DAS**	**90 DAS**	**ET (Plant** ^−1^ **)**	**SL** **(cm)**	**Spikelet spike** ^−1^	**Grain spike** ^−1^	**Test weight**				
**Without Zn**															
Control (N_0_P_0_K_0_)	18.23^e^	32.51^e^	63.61^e^	0.27^e^	1.45^d^	4.52^e^	3.33^e^	8.04^e^	17.42^e^	28.32^b^	37.82^a^	3.16^e^	5.01^e^	8.17^e^	39.31^a^
RDF (N_120_P_60_K_60_)	20.45^d^	35.61^d^	70.40^d^	0.32^d^	1.52^c^	4.78^d^	3.54^d^	8.78^d^	18.19^d^	31.84^a^	38.33^a^	3.96^d^	6.10^d^	10.06^d^	39.36^a^
RDF + *Bacillus altitudinis* AJW-3	21.64^abc^	36.73^abc^	74.59^bc^	0.34^b^	1.61^ab^	5.04^bc^	3.76^ab^	9.15^ab^	19.08^b^	32.32^a^	38.37^a^	4.23^ab^	6.41^ab^	10.64^ab^	39.70^a^
RDF + *Bacillus subtilis* ABW-30	21.40^c^	36.08^c^	72.39^cd^	0.34^b^	1.58^b^	4.88^c^	3.68^bc^	8.99^bc^	18.78^bc^	32.11^a^	38.28^a^	4.14^c^	6.31^c^	10.45^c^	39.63^a^
RDF + *Bacillus megaterium* CHW-22	21.94^a^	37.58^a^	78.02^a^	0.35^a^	1.68^a^	5.27^a^	3.84^a^	9.32^a^	19.87^a^	32.55^a^	38.48^a^	4.32^a^	6.49^a^	10.81^a^	39.95^a^
RDF + *Bacillus licheniformis* MJW-38	21.81^ab^	37.14^ab^	76.47^ab^	0.35^a^	1.64^ab^	5.18^ab^	3.82^a^	9.22^ab^	19.43^ab^	32.43^a^	38.41^a^	4.28^a^	6.49^a^	10.77^a^	39.73^a^
RDF + *Brevibacillus borstelensis* CHW-2	21.31^c^	35.94^c^	71.96^cd^	0.33^c^	1.56^bc^	4.80^cd^	3.64^c^	8.90^c^	18.68^c^	32.04^a^	38.36^a^	4.09^c^	6.34^c^	10.43^c^	39.41^a^
RDF + *Bacillus xiamenensis* BLW-7	21.54^bc^	36.25^bc^	73.39^c^	0.34^b^	1.60^ab^	4.98^bc^	3.72^b^	9.06^bc^	18.89^bc^	32.18^a^	38.33^a^	4.18^ab^	6.35^ab^	10.53^ab^	39.70^a^
**Zn Applied**															
Control (N_0_P_0_K_0_)	19.46^d^	34.2^d^	66.41^f^	0.28^d^	1.53^b^	4.65^e^	3.50^e^	8.41^e^	18.26^d^	29.51^b^	39.60^a^	3.35^e^	4.99^e^	8.34^e^	40.16^a^
RDF (N_120_P_60_K_60_)	21.78^c^	37.6^c^	73.71^e^	0.33^c^	1.63^ab^	4.90^d^	3.70^d^	9.20^d^	19.08^cd^	33.27^a^	40.21^a^	4.19^d^	6.14^d^	10.33^d^	40.54^a^
RDF + *Bacillus altitudinis* AJW-3	22.88^ab^	38.9^ab^	78.47^bc^	0.36^ab^	1.71^ab^	5.27^ab^	4.00^ab^	9.66^ab^	20.15^abc^	34.00^a^	40.48^a^	4.49^ab^	6.51^ab^	11.00^ab^	40.82^a^
RDF + *Bacillus subtilis* ABW-30	22.72^b^	38.1^b^	75.94^cde^	0.35^b^	1.68^ab^	5.09^ab^	3.90^bc^	9.47^bcd^	19.74^bc^	33.68^a^	40.27^a^	4.38^c^	6.38^c^	10.76^c^	40.74^a^
RDF + *Bacillus megaterium* CHW-22	23.12^a^	39.9^a^	82.23^a^	0.37^a^	1.80^a^	5.53^a^	4.10^a^	9.87^a^	21.04^a^	34.41^a^	40.75^a^	4.60^a^	6.61^a^	11.21^a^	41.04^a^
RDF + *Bacillus licheniformis* MJW-38	23.03^ab^	39.4^ab^	80.52^ab^	0.36^ab^	1.75^a^	5.42^a^	4.00^b^	9.75^ab^	20.54^ab^	34.21^a^	40.60^a^	4.57^a^	6.57^a^	11.14^a^	41.04^a^
RDF + *Brevibacillus borstelensis* CHW-2	22.67^b^	38.0^bc^	75.49^de^	0.35^b^	1.66^c^	5.00^c^	3.80^c^	9.35^cd^	19.61^bc^	33.55^a^	40.28^a^	4.38^c^	6.32^c^	10.70^c^	40.72^a^
RDF + *Bacillus xiamenensis* BLW-7	22.84^ab^	38.3^b^	77.06^cd^	0.35^b^	1.69^ab^	5.21^ab^	3.90^bc^	9.55^bc^	19.89^abc^	33.82^a^	40.36^a^	4.43^ab^	6.44^ab^	10.87^ab^	40.77^a^

RDF: recommended dose of fertilizers: 120 kg N ha^−1^, 60 kg P ha^−1^, and 60 kg K ha^−1^, DMA: dry matter accumulation, GY: grain yield, SY: straw yield, and BY: biological yield: HI: Harvesting Index, ET: effective tiller, SL: spike length, and DAS: days after sowing. Data with different letters show significant difference in column data in randomized block design test at *p* < 0.05 under Duncan's multiple range test.

Similarly, a significantly higher number of effective tiller plant^−1^ (3.84), spike length (9.32c m), spikelet spike^−1^ (19.87), number of grain spike^−1^ (32.55), and test weight (38.48 g) were recorded in the plants inoculated with *B. megaterium* CHW-22 without zinc phosphate followed by *B. licheniformis* MJW-38 (number of effective tiller plant^−1^−3.82, spike length-−9.22 cm, spikelet spike^−1^−19.43, number of grain spike^−1^−32.43, and test weight-−38.41 g) and *B. altitudinis* AJW-3 (number of effective tiller plant^−1^−3.76, spike length-−9.15 cm, spikelet spike^−1^−19.08, number of grain spike^−1^−32.32, and test weight-−38.37 g) as compared to other inoculants, RDF alone, and untreated absolute control. A similar pattern was reported in the plants amended with zinc phosphate and inoculated with microbial inoculants. However, these values are slightly higher as compared to treatments without zinc phosphate (Table 2).

Furthermore, grain yield (g pot^−1^), straw yield (g pot^−1^), biological yield (g pot^−1^), and harvest index (%) were also recorded in the plants inoculated with selected microbial inoculants supplemented with and without zinc phosphate under nethouse conditions. The results indicated that maximum grain yield (4.32 and 4.28 g pot^−1^), straw yield (6.49 and 6.49 g pot^−1^), biological yield (10.81 and 10.77 g pot^−1^), and harvest index (39.95 and 39.73%) were recorded in the plants inoculated with *B. megaterium* CHW-22 and *B. licheniformis* MJW-38, respectively, without zinc phosphate followed by *B. altitudinis* AJW-3 (grain yield-−4.23 g pot^−1^, straw yield-−6.41 g pot^−1^, biological yield-−10.64 g pot^−1^, and harvest index 39.70%) under nethouse conditions (Table 2). A more or less similar trend was recorded in the plant grown with zinc phosphate. However, these values were slightly higher in the case of zinc phosphate-amended plants (Table 2).

### Effect of microbial inoculation on SOC, available nutrients, and microbial activity in rhizosphere soil

Unlike plant growth and yield attributes, microbial inoculation has a positive impact on SOC, available nutrients, and microbial activity in rhizosphere soil, which is further increased after adding zinc phosphate in general. When bioprimed seeds were sown in the pots, potential zinc-solubilizing plant growth-promoting bacteria reached the rhizosphere soil. The results obtained from the nethouse experiments showed that inoculation of *B. megaterium* CHW-22 has an impact on the percent soil organic carbon in the rhizosphere soil as compared to other inoculants. However, the differences were not significant. Similarly, these values were slightly higher in the soil amended with zinc phosphate and inoculated with rhizobacterial inoculants, RDF, and absolute control as compared to unamended soil (without zinc phosphate). Moreover, the trend was more or less similar (Table 3). Furthermore, inoculation of these bacteria significantly increased the available N, P, and K content in the rhizosphere soil amended with and without zinc phosphate under the nethouse conditions at harvest. As reported earlier, maximum available N (159.80 kg ha^−1^), P (16.32 kg ha^−1^), and K (222.60 kg ha^−1^) content was reported in the rhizosphere soil of plants inoculated with *B. megaterium* CHW-22 without zinc phosphate followed by *B. licheniformis* MJW-38 (N: 157.60 kg ha^−1^, P: 16.21 kg ha^−1^ and K: 220.40 kg ha^−1^) and *B. altitudinis* AJW-3 (N: 155.30 kg ha^−1^, P: 16.14 kg ha^−1^ and K: 219.20 kg ha^−1^). However, the least content was reported in the absolute control (N: 141.80 kg ha^−1^, P: 13.92 kg ha^−1^ and K: 204.30 kg ha^−1^) and RDF (N: 150.40 kg ha^−1^, P: 15.32 kg ha^−1^ and K: 208.30 kg ha^−1^) under the nethouse conditions (Table 3). A more or less similar pattern was recorded in the rhizosphere soil amended with zinc phosphate with higher values (Table 3).

**Table 3:** Effect of zinc-solubilizing plant growth-promoting rhizobacteria on organic carbon, available nutrients, and microbial activity in rhizosphere soil of wheat under pot experiment.

**Treatment**	**SOC**	**N**	**P**	**K**	**Fe**	**Zn**	**Cu**	**Mn**	**DHA**	**APA**	**FDA**	**SMBC**
	**%**	**kg ha** ^−1^	**kg ha** ^−1^	**kg ha** ^−1^	μ**g g**^−1^	μ**g g**^−1^	μ**g g**^−1^	μ**g g**^−1^	μ**g TPF g**^−1^ **soil 24 h**^−1^	μ**g pNPg**^−1^ **soil h**^−1^	μ**g FLR g**^−1^ **soil hr**^−1^	μ**g g**^−1^ **soil**
**Without Zn**												
Control (N_0_P_0_K_0_)	0.394^a^	141.8^e^	13.92^f^	204.30^f^	4.21^e^	0.57^f^	1.72^e^	5.12^f^	112.80^e^	78.34^f^	14.32^e^	104.4^e^
RDF (N_120_P_60_K_60_)	0.404^a^	150.4^d^	15.32^e^	208.30^e^	4.34^d^	0.61^e^	1.77^d^	5.19^e^	119.60^d^	82.45^e^	15.48^d^	109.2^d^
RDF + *Bacillus altitudinis* AJW-3	0.419^a^	155.3^bc^	16.14^ab^	219.20^abc^	4.48^b^	0.85^bc^	1.83^ab^	5.39^abc^	129.50^abc^	94.88^bc^	18.32^bc^	128.4^ab^
RDF + *Bacillus subtilis* ABW-30	0.412^a^	152.7^c^	15.88^d^	215.60^cd^	4.39^c^	0.78^d^	1.80^c^	5.32^c^	126.20^c^	92.32^cd^	17.58^cd^	123.8^bc^
RDF + *Bacillus megaterium* CHW-22	0.424^a^	159.8^a^	16.32^a^	222.60^a^	4.56^a^	0.92^a^	1.86^a^	5.46^a^	134.80^a^	98.48^a^	21.46^a^	132.7^a^
RDF + *Bacillus licheniformis* MJW-38	0.421^a^	157.6^ab^	16.21^ab^	220.40^ab^	4.52^ab^	0.88^ab^	1.85^a^	5.42^ab^	132.30^ab^	96.36^ab^	20.32^ab^	129.6^ab^
RDF + *Brevibacillus borstelensis* CHW-2	0.409^a^	151.5^cd^	15.78^ed^	213.40^d^	4.36^cd^	0.76^d^	1.80^c^	5.25^d^	125.10^c^	89.85^d^	16.48^cde^	118.8^c^
RDF + *Bacillus xiamenensis* BLW-7	0.415^a^	154.9^bc^	16.08^c^	217.80^bc^	4.44^bc^	0.82^c^	1.82^ab^	5.34^bc^	127.90^bc^	93.52^cd^	17.56^cd^	125.6^b^
**Zn Applied**												
Control (N_0_P_0_K_0_)	0.412^a^	150.3^e^	12.64^e^	206.82^f^	4.46^e^	0.68^f^	1.78^d^	5.38^f^	120.20^d^	83.04^e^	15.04^g^	109.62^f^
RDF (N_120_P_60_K_60_)	0.423^a^	160.8^d^	14.83^d^	214.13^e^	4.60^d^	0.74^e^	1.84^cd^	5.42^e^	126.30^c^	87.88^d^	16.56^f^	115.75^e^
RDF + *Bacillus altitudinis* AJW-3	0.438^a^	164.3^bc^	15.62^ab^	226.87^abc^	4.70^bc^	1.11^c^	1.90^ab^	5.68^ab^	136.13^ab^	100.57^bc^	19.42^bc^	134.82^bc^
RDF + *Bacillus subtilis* ABW-30	0.427^a^	162.5^bc^	15.08^c^	222.50^cd^	4.61^c^	1.00^d^	1.88^bc^	5.58^c^	132.84^bc^	97.58^c^	18.72^d^	131.23^cd^
RDF + *Bacillus megaterium* CHW-22	0.444^a^	170.7^a^	16.02^a^	230.84^a^	4.83^a^	1.25^a^	1.95^a^	5.73^a^	142.35^a^	105.87^a^	23.28^a^	143.32^a^
RDF + *Bacillus licheniformis* MJW-38	0.441^a^	166.7^ab^	15.71^ab^	228.33^ab^	4.79^ab^	1.17^b^	1.94^a^	5.72^a^	138.91^ab^	103.88^ab^	21.95^ab^	138.67^ab^
RDF + *Brevibacillus borstelensis* CHW-2	0.429^a^	160.^4c^	15.03^c^	219.59^d^	4.60^d^	0.99^ed^	1.86^c^	5.52^d^	132.11^bc^	95.96^c^	17.80^e^	127.12^d^
RDF + *Bacillus xiamenensis* BLW-7	0.431^a^	163.6^bc^	15.09^c^	225.21^bc^	4.68^bc^	1.07^cd^	1.90^ab^	5.64^bc^	133.83^bc^	100.41^bc^	19.03^c^	135.65^bc^

RDF, recommended dose of fertilizers; 120 kg N ha^−1^, 60 kg P ha^−1^, and 60 kg K ha^−1^; SOC, soil organic carbon; DHA, dehydrogenase activity; APA, alkaline phosphatase activity; FDA, fluorescein diacetate activity; SMBC, soil microbial biomass carbon; TPF, triphenylformazan; pNP, p-nitrophenol phosphatase; FLR, fluorescein. Data with different letters show significant difference in column data in randomized block design test at *p* < 0.05 under Duncan's multiple range test.

When comparing the effect of inoculation on the availability of micronutrients, i.e., Fe, Zn, Cu, and Mn, maximum content was reported in the plant rhizosphere inoculated with *B. megaterium* CHW-22 (4.56, 0.92, 1.86, and 5.46 μg g^−1^, respectively) without zinc phosphate followed by *B. licheniformis* (4.52, 0.88, 1.85, and 5.42 μg g^−1^, respectively) and *B. altitudinis* (4.48, 0.85, 1.83, and 5.39 μg g^−1^, respectively). A similar trend was observed in the soil amended with zinc phosphate (Table 3). The results clearly revealed that maximum DHA (134.80 μg TPF g^−1^ soil 24 h^−1^), APA (98.48 μg pNPg^−1^ soil h^−1^), FDA (21.46 μg FLR g^−1^ soil hr^−1^), and SMBC (132.70 μg g^−1^ soil) were recorded in the rhizosphere of plants inoculated with *B. megaterium* CHW-22 without zinc phosphate followed by *B. licheniformis* MJW-38 and *B. altitudinis* AJW-3 as compared to other inoculants and absolute control plants. Moreover, a similar pattern with slightly higher values was recorded in the rhizosphere of plants inoculated with rhizobacteria and amended with zinc phosphate at harvest (Table 3).

### Effect of microbial inoculation on nutritional content in wheat

Microbial inoculation significantly affects nutritional content in wheat amended with and without zinc phosphate under pot experiments. The results clearly revealed that *B. megaterium* CHW-22 was the most potential inoculant, in general, and it has a significant impact on the nutritional biofortification in wheat under nethouse conditions. It was further observed that maximum N, P, and K content in the wheat grain and straw was recorded in the treatment RDF + *B. megaterium* CHW-22 and unamended zinc phosphate followed by *B. licheniformis* MJW-38 and *B. altitudinis* AJW-3 as compared to other inoculants and untreated control plants. When evaluating the micronutrients, especially Zn, maximum accumulation of Zn was reported in the grain and straw obtained from the wheat plants bioprimed with *B. megaterium* CHW-22 (46.44 and 33.64 μg g^−1^, respectively) followed by *B. licheniformis* MJW-38 (45.28 and 32.98 μg g^−1^, respectively) and *B. altitudinis* AJW-3 (43.11 and 31.38 μg g^−1^, respectively) as compared to other treatments. Similarly, maximum Fe, Cu, and Mn were reported in the grain and straw obtained from the wheat plants bioprimed with *B. megaterium* CHW-22 followed by *B. licheniformis* MJW-38 and *B. altitudinis* AJW-3 as compared to other treatments. However, the least concentration of macronutrients (N, P, and K) and micronutrients including Zn (Zn, Fe, Cu, and Mn) was reported in the untreated absolute control and RDF alone amended plants without zinc phosphate (Table 4). A more or less similar pattern was recorded in the case of zinc phosphate-amended plants inoculated with selected strains of rhizobacterial inoculants (Table 4).

**Table 4:** Effect of zinc-solubilizing plant growth-promoting rhizobacteria on nutrition concentration in wheat under pot experiment.

**Treatment**	**N (%)**		**P (%)**		**K (%)**		**Zn (**μ**g g**^**−1**^**)**		**Fe (**μ**g g**^**−1**^**)**		**Cu (**μ**g g**^**−1**^**)**		**Mn (**μ**g g**^**−1**^**)**	
	**Grain**	**Straw**	**Grain**	**Straw**	**Grain**	**Straw**	**Grain**	**Straw**	**Grain**	**Straw**	**Grain**	**Straw**	**Grain**	**Straw**
**Without Zn**														
Control (N_0_P_0_K_0_)	1.76^e^	0.46^d^	0.31^e^	0.11^b^	0.44^d^	1.47^d^	34.12^g^	25.32^e^	76.93^e^	245.43^f^	10.71^d^	7.48^f^	25.04^f^	33.80^g^
RDF (N_120_P_60_K_60_)	1.84^d^	0.50^c^	0.33^d^	0.11^b^	0.49^c^	1.53^c^	36.23^f^	26.88^d^	80.46^de^	255.37^e^	10.79^c^	7.54^e^	25.28^e^	34.18^f^
RDF + *Bacillus altitudinis* AJW-3	1.94^ab^	0.54^ab^	0.38^ab^	0.12^a^	0.54^ab^	1.60^ab^	43.11^bc^	31.38^ab^	88.94^abc^	275.96^c^	11.02^b^	7.73^c^	26.12^ab^	35.38^bc^
RDF + *Bacillus subtilis* ABW-30	1.87^bc^	0.52^bc^	0.36^bc^	0.12^a^	0.53^abc^	1.58^abc^	40.45^de^	29.19^bc^	85.36^cd^	271.67^cd^	10.88^bc^	7.61^d^	25.84^c^	34.98^d^
RDF + *Bacillus megaterium* CHW-22	1.98^a^	0.57^a^	0.39^a^	0.12^a^	0.55^a^	1.62^a^	46.44^a^	33.64^a^	93.27^a^	287.51^a^	11.20^a^	8.45^a^	26.36^a^	35.72^a^
RDF + *Bacillus licheniformis* MJW-38	1.96^ab^	0.56^a^	0.38^ab^	0.12^a^	0.54^ab^	1.62^a^	45.28^ab^	32.98^a^	91.92^ab^	280.33^b^	11.17^a^	7.93^b^	26.28^a^	35.54^ab^
RDF + *Brevibacillus borstelensis* CHW-2	1.86^cd^	0.52^bc^	0.35^c^	0.12^a^	0.52^bc^	1.57^bc^	40.28^e^	29.00^c^	85.03^cd^	268.66^d^	10.88^bc^	7.60^d^	25.45^d^	34.62^e^
RDF + *Bacillus xiamenensis* BLW-7	1.89^bc^	0.54^ab^	0.37^abc^	0.12^a^	0.54^ab^	1.60^ab^	42.92^cd^	31.08^abc^	86.84^bc^	274.25^c^	10.97^b^	7.61^d^	25.98^bc^	35.22^c^
**Zn Applied**														
Control (N_0_P_0_K_0_)	1.83^c^	0.49^d^	0.33^e^	0.12^b^	0.48^d^	1.55^d^	37.87^g^	27.60^e^	80.77^e^	258.94^g^	11.46^e^	8.03^d^	27.04^f^	35.85^e^
RDF (N_120_P_60_K_60_)	1.91^bc^	0.54^c^	0.36^d^	0.12^b^	0.53^c^	1.64^c^	40.58^f^	29.57^d^	84.64^d^	267.63^f^	11.65^d^	8.11^c^	27.05^e^	36.57^d^
RDF + *Bacillus altitudinis* AJW-3	2.02^ab^	0.58^ab^	0.40^abc^	0.13^a^	0.57^abc^	1.70^ab^	50.87^bc^	36.71^b^	94.10^abc^	290.59^c^	11.79^c^	8.27^bc^	28.17^bc^	38.21^bc^
RDF + *Bacillus subtilis* ABW-30	1.97^abc^	0.57^abc^	0.39^bc^	0.13^a^	0.57^abc^	1.68^abc^	47.33^de^	33.86^bc^	91.32^bc^	286.07^de^	11.69^d^	8.21^bc^	27.61^d^	38.03^c^
RDF + *Bacillus megaterium* CHW-22	2.07^a^	0.60^a^	0.43^a^	0.14^a^	0.61^a^	1.73^a^	55.73^a^	40.70^a^	97.75^a^	302.46^a^	12.15^a^	8.52^a^	28.73^a^	39.29^a^
RDF + *Bacillus licheniformis* MJW-38	2.03^ab^	0.60^a^	0.41^ab^	0.13^a^	0.58^ab^	1.71^a^	53.88^ab^	39.88^a^	96.42^ab^	295.47^b^	11.95^b^	8.36^ab^	28.38^ab^	38.56^b^
RDF + *Brevibacillus borstelensis* CHW-2	1.95^abc^	0.55^bc^	0.38^c^	0.13^a^	0.57^abc^	1.68^abc^	46.72^e^	32.93^c^	91.20^bc^	285.58^e^	11.65^d^	8.13^c^	27.44^d^	38.01^c^
RDF + *Bacillus xiamenensis* BLW-7	1.99^ab^	0.58^ab^	0.40^abc^	0.13^a^	0.57^abc^	1.70^ab^	50.43^cd^	36.67^b^	91.36^bc^	288.51^cd^	11.74^cd^	8.21^bc^	27.80^c^	38.04^c^

RDF: recommended dose of fertilizers: 120 kg N ha^−1^, 60 kg P ha^−1^, and 60 kg K ha^−1^. Data with different letters show significant difference in column data in randomized block design test at *p* < 0.05 under Duncan's multiple range test.

### Evaluation of microbial inoculants under field conditions

#### Plant growth and yield attributes

Inoculation of selected potential strains of zinc-solubilizing rhizobacteria significantly increased the plant height, dry matter accumulation, yield and yield attributes, and harvest index as compared to the absolute control plants and RDF (alone) applied plants under field conditions. Similar to the observations under the pot experiment, *B. megaterium* CHW-22 was the best-performing strain followed by *B. licheniformis* MJW-38 and *B. altitudinis* AJW-3 in general. From the results of the present investigation, it was clearly noticed that maximum plant height was recorded for *B. megaterium* CHW-22-inoculated plants followed by *B. licheniformis* MJW-38 and *B. altitudinis* AJW-3-inoculated plants at 30, 60, and 90 DAS as compared to other treatments under field conditions. *B. subtilis* ABW-30, *B. borstelensis* CHW-2, and *B. xiamenensis* BLW-7 showed minor differences (Table 5). The seed biopriming with *B. megaterium* CHW-22 significantly increased the dry matter accumulation followed by *B. licheniformis* MJW-38 and *B. altitudinis* AJW-3 as compared to absolute control plants and RDF-applied plants. All the other strains significantly increased dry matter as compared to the absolute control, but this increase was significantly less when compared to *B. megaterium* CHW-22 treated plants at 30, 60, and 90 DAS under field conditions (Table 5). The results obtained from field experiments include the maximum number of effective tiller m^−2^ (442.6), spike length (11.24 cm), spikelet spike^−1^ (24.69), number of grain spike^−1^ (44.98), and test weight (40.04 g) being recorded in the plants bioprimed with *B. megaterium* CHW-22 without zinc phosphate followed by *B. licheniformis* MJW-38 and *B. altitudinis* AJW-3 as compared to other inoculants, RDF alone, and untreated absolute control. A more or less similar trend was reported in the plants amended with zinc phosphate and inoculated with microbial inoculants. However, these values are slightly higher as compared to the treatments without zinc phosphate (Table 5). Similarly, seed biopriming has a positive impact on grain yield (q ha^−1^), straw yield (q ha^−1^), biological yield (q ha^−1^), and harvest index (%) under field conditions. Maximum grain yield (46.42 q ha^−1^), straw yield (68.71 q ha^−1^), biological yield (115.13 q ha^−1^), and harvest index (40.32 %) were recorded in the plants inoculated with *B. megaterium* CHW-22 without zinc phosphate as compared to other inoculants under field conditions. There was a minor difference in the harvest index but a non-significant increase was observed in the harvest index (%) of plants inoculated with either the inoculants or the absolute control and RDF alone applied plants. A more or less similar trend was recorded in the grain yield (q ha^−1^), straw yield (q ha^−1^), biological yield (q ha^−1^), and harvest index (%) in the plants bioprimed with selected rhizobacterial strains and amended with zinc phosphate. However, these values were slightly higher in the case of zinc phosphate-amended plants under field conditions (Table 5).

**Table 5:** Effect of zinc-solubilizing plant growth-promoting rhizobacteria on growth, yield attributes, and yield of wheat under field experiment.

**Treatments**	**Plant height (cm)**			**DMA (g m** ^**−2**^ **)**			**Yield attribute**					**GY (q ha^−1^)**	**SY (q ha^−1^)**	**BY (q ha^−1^)**	**HI (%)**
	**30 DAS**	**60 DAS**	**90 DAS**	**30 DAS**	**60 DAS**	**90 DAS**	**ET (M^−2^)**	**SL (cm)**	**Spikelet spike** ^−1^	**Grain spike** ^−1^	**Test weight**				
**Without Zn**															
Control (N_0_P_0_K_0_)	21.09^d^	39.12^e^	77.48^e^	17.66^d^	116.4^c^	390.3^d^	391.2^c^	9.88^e^	21.32^e^	39.78^e^	39.43^b^	34.87^e^	53.23^d^	88.10^c^	39.58^a^
RDF (N_120_P_60_K_60_)	24.78^c^	42.40^d^	85.44^d^	21.12^c^	124.5^b^	428.8^c^	425.9^b^	10.77^d^	22.66^d^	42.12^d^	39.84^a^	44.78^d^	67.23^c^	112.01^b^	39.98^a^
RDF + *Bacillus altitudinis* AJW-3	26.60^ab^	43.42^b^	89.44^b^	22.78^a^	129.8^ab^	454.8^ab^	430.7^ab^	11.12^ab^	23.89^ab^	43.46^bc^	39.98^a^	46.18^ab^	68.64^ab^	114.82^a^	40.22^a^
RDF + *Bacillus subtilis* ABW-30	25.94^b^	42.80^c^	87.32^c^	22.32^ab^	127.0^b^	439.7^bc^	429.7^ab^	10.98^bc^	23.20^bc^	42.88^c^	39.89^a^	45.94^bc^	68.42^b^	114.36^a^	40.17^a^
RDF + *Bacillus megaterium* CHW-22	27.52^a^	44.26^a^	92.99^a^	22.96^a^	132.4^a^	472.4^a^	442.6^a^	11.24^a^	24.69^a^	44.98^a^	40.04^a^	46.42^a^	68.71^a^	115.13^a^	40.32^a^
RDF + *Bacillus licheniformis* MJW-38	27.27^a^	43.79^ab^	91.36^ab^	22.82^a^	131.6^a^	465.6^a^	438.5^a^	11.20^a^	23.97^ab^	43.82^b^	39.99^a^	46.24^ab^	68.56^ab^	114.80^a^	40.28^a^
RDF + *Brevibacillus borstelensis* CHW-2	25.73^bc^	42.74^c^	87.12^c^	22.08^b^	126.5^ab^	437.3^bc^	424.4^b^	10.89^c^	23.01^c^	42.69^cd^	39.87^a^	45.72^c^	68.27^bc^	113.99^ab^	40.11^a^
RDF + *Bacillus xiamenensis* BLW-7	26.52^ab^	42.89^c^	88.32^bc^	22.45^ab^	128.4^ab^	446.2^b^	430.8^ab^	11.04^b^	23.43^b^	43.35^bc^	39.92^a^	46.08^b^	68.58^ab^	114.66^a^	40.19^a^
**Zn Applied**															
Control (N_0_P_0_K_0_)	22.3^e^	40.8^c^	80.66^e^	18.51^d^	123.4^c^	405.12^d^	409.6^d^	10.32^e^	22.30^e^	41.29^e^	40.18^b^	36.13^e^	54.51^c^	90.64^d^	39.86^a^
RDF (N_120_P_60_K_60_)	25.6^d^	44.6^b^	89.03^d^	22.18^c^	130.1^bc^	440.95^c^	440.7^c^	11.35^d^	23.65^d^	43.80^d^	40.63^a^	46.48^d^	68.53^bc^	115.01^c^	40.42^a^
RDF + *Bacillus altitudinis* AJW-3	27.8^b^	46.0^ab^	94.81^b^	23.95^ab^	137.6^ab^	475.76^ab^	458.8^ab^	11.80^ab^	24.59^bc^	45.85^ab^	41.02^a^	48.30^ab^	70.25^a^	118.56^a^	40.74^a^
RDF + *Bacillus subtilis* ABW-30	26.9^c^	45.1^ab^	91.08^c^	23.50^b^	135.3^ab^	454.05^b^	450.1^b^	11.65^bc^	24.13^cd^	44.68^bc^	40.80^a^	47.87^bc^	70.00^b^	117.87^ab^	40.61^a^
RDF + *Bacillus megaterium* CHW-22	28.9^a^	46.9^a^	97.55^a^	24.31^a^	140.4^a^	495.55^a^	467.9^a^	11.90^a^	25.65^a^	47.50^a^	41.20^a^	48.79^a^	70.19^a^	118.98^a^	41.01^a^
RDF + *Bacillus licheniformis* MJW-38	28.6^a^	46.9^a^	97.46^a^	24.22^a^	139.5^a^	487.48^a^	462.6^a^	11.83^ab^	24.89^b^	46.10^ab^	41.07^a^	48.51^ab^	70.14^a^	118.64^a^	40.88^a^
RDF + *Brevibacillus borstelensis* CHW-2	26.7^cd^	44.9^ab^	90.87^cd^	23.43^b^	134.1^b^	450.46^b^	449.2^b^	11.50^c^	24.02^cd^	44.48^c^	40.74^a^	47.50^c^	69.76^b^	117.26^b^	40.51^a^
RDF + *Bacillus xiamenensis* BLW-7	27.0^c^	45.2^ab^	92.21^c^	23.86^ab^	136.2^ab^	469.28^ab^	456.2^ab^	11.75^b^	24.37^c^	45.13^b^	40.91^a^	48.11^b^	70.17^ab^	118.28^a^	40.67^a^

RDF: recommended dose of fertilizers: 120 kg N ha^−1^, 60 kg P ha^−1^, and 60 kg K ha-1. DMA, dry matter accumulation; GY, grain yield; SY, straw yield; and BY, biological yield; HI, Harvesting index; ET, effective tiller; SL, spike length; and DAS, days after sowing. Data with different letters show significant difference in column data in randomized block design test at *p* < 0.05 under Duncan's multiple range test.

### Effect of microbial inoculation on SOC, available nutrients, and microbial activity in rhizosphere soil

In line with the results obtained under nethouse conditions, rhizobacterial inoculation was observed to have a significant impact on SOC, available nutrients, and microbial activity in rhizosphere soil under field conditions too. These properties were further increased after adding zinc phosphate in general. Similar to the nethouse results, it was observed that seed bioprimed with *B. megaterium* CHW-22 has an impact on soil organic carbon (%) in the rhizosphere soil as compared to other treatments. However, the differences were not significant statistically. Similarly, these values were slightly higher in the soil amended with zinc phosphate and inoculated with rhizobacterial strains, RDF alone, and absolute control as compared to unamended soil (without zinc phosphate) under field conditions. However, the trend was more or less similar (Table 6). It was further observed that inoculation of rhizobacterial strains significantly increased the availability of macronutrients (N, P, and K) and micronutrients (Fe, Zn, Cu, and Mn) in the wheat rhizosphere soil amended with and without zinc phosphate under field conditions at harvest. Among the different inoculants, the maximum availability of macronutrients (N, P, and K) and micronutrients (Fe, Zn, Cu, and Mn) was recorded in the rhizosphere soil of wheat inoculated with *B. megaterium* CHW-22 amended with and without zinc phosphate under field conditions followed by *B. licheniformis* MJW-38- and *B. altitudinis* AJW-3-inoculated plant rhizosphere soil as compared to other inoculants, RDF alone, and untreated absolute control plants. However, the least available nutrient was reported in the rhizosphere of absolute control (Table 6). The results showed that maximum DHA (141.81 μg TPF g^−1^ soil 24 h^−1^), APA (104.19 μg pNPg^−1^ soil h^−1^), FDA (22.88 μg FLR g^−1^ soil hr^−1^), and SMBC (140.66 μg g^−1^ soil) were reported in the rhizosphere of plants inoculated with *B. megaterium* CHW-22 without zinc phosphate followed by *B. licheniformis* MJW-38 and *B. altitudinis* AJW-3 as compared to other treatments. Moreover, a similar pattern with slightly higher values was recorded for DHA, APA, FDA, and SMBC in the rhizosphere of plants inoculated with rhizobacteria and amended with zinc phosphate at harvest (Table 6).

**Table 6:** Effect of zinc-solubilizing plant growth-promoting rhizobacteria on organic carbon, available nutrients, and microbial activity in rhizosphere soil of wheat under field experiment.

**Treatment**	**SOC**	**N**	**P**	**K**	**Fe**	**Zn**	**Cu**	**Mn**	**DHA**	**APA**	**FDA**	**SMBC**
	**%**	**kg ha^−1^**	**kg ha^−1^**	**kg ha^−1^**	**μg g^−1^**	**μg g^−1^**	**μg g^−1^**	**μg g^−1^**	**μg TPF g^−1^ soil 24 h^−1^**	**μg pNPg^−1^ soil h^−1^**	**μg FLR g^−1^ soil hr^−1^**	**μg g^−1^ soil**
**Without Zn**												
Control (N_0_P_0_K_0_)	0.387^a^	134.68^e^	13.99^e^	198.40^c^	4.14^d^	0.55^e^	1.69^c^	5.04^e^	116.86^e^	81.71^e^	15.11^e^	109.41^f^
RDF (N_120_P_60_K_60_)	0.399^a^	142.32^d^	15.41^d^	202.80^b^	4.21^c^	0.61^d^	1.76^b^	5.07^d^	124.03^d^	86.16^d^	16.36^d^	114.66^e^
RDF + *Bacillus altitudinis* AJW-3	0.412^a^	147.55^b^	16.31^ab^	212.80^a^	4.38^ab^	0.83^b^	1.81^ab^	5.16^ab^	135.33^b^	99.91^b^	19.47^b^	135.72^b^
RDF + *Bacillus subtilis* ABW-30	0.407^a^	145.66^bc^	16.13^bc^	209.30^ab^	4.28^bc^	0.78^c^	1.79^ab^	5.12^b^	131.12^c^	96.84^c^	18.63^bc^	130.49^cd^
RDF + *Bacillus megaterium* CHW-22	0.418^a^	158.83^a^	16.42^a^	216.40^a^	4.48^a^	0.88^a^	1.87^a^	5.21^a^	141.81^a^	104.19^a^	22.88^a^	140.66^a^
RDF + *Bacillus licheniformis* MJW-38	0.414^a^	148.48^b^	16.39^a^	214.60^a^	4.42^a^	0.86^ab^	1.84^a^	5.18^a^	141.56^a^	101.66^ab^	21.62^ab^	137.12^ab^
RDF + *Brevibacillus borstelensis* CHW-2	0.404^a^	144.98^c^	15.96^c^	207.60^ab^	4.26^bc^	0.76^cd^	1.78^ab^	5.09^c^	129.85^cd^	94.07^cd^	17.45^c^	124.98^d^
RDF + *Bacillus xiamenensis* BLW-7	0.409^a^	146.32^bc^	16.21^b^	211.20^a^	4.33^b^	0.81^bc^	1.80^ab^	5.14^ab^	133.27^bc^	98.38^bc^	18.54^bc^	132.51^c^
**Zn Applied**												
Control (N_0_P_0_K_0_)	0.399^a^	138.05^e^	13.65^e^	203.56^d^	4.27^d^	0.65^f^	1.74^c^	5.23^d^	119.78^e^	84.00^f^	15.65^e^	112.69^f^
RDF (N_120_P_60_K_60_)	0.412^a^	146.30^d^	14.99^d^	208.48^c^	4.38^c^	0.73^e^	1.82^b^	5.27^c^	127.75^d^	88.66^e^	16.98^d^	118.33^e^
RDF + *Bacillus altitudinis* AJW-3	0.425^a^	152.27^b^	15.78^ab^	220.25^a^	4.55^ab^	1.05^b^	1.88^ab^	5.38^ab^	140.06^b^	103.41^b^	20.33^b^	140.88^b^
RDF + *Bacillus subtilis* ABW-30	0.422^a^	150.76^bc^	15.58^bc^	216.00^ab^	4.43^bc^	0.95^d^	1.85^ab^	5.32^b^	135.45^c^	99.85^c^	19.40^bc^	135.05^c^
RDF + *Bacillus megaterium* CHW-22	0.434^a^	164.87^a^	15.94^a^	224.62^a^	4.66^a^	1.14^a^	1.95^a^	5.45^a^	146.78^a^	108.36^a^	23.97^a^	146.29^a^
RDF + *Bacillus licheniformis* MJW-38	0.427^a^	153.97^b^	15.88^a^	222.54^a^	4.59^a^	1.10^ab^	1.92^a^	5.41^a^	146.66^a^	105.52^ab^	22.62^ab^	142.46^ab^
RDF + *Brevibacillus borstelensis* CHW-2	0.417^a^	149.91^c^	15.44^c^	213.83^b^	4.41^bc^	0.92^de^	1.84^ab^	5.29^bc^	134.01^cd^	96.90^d^	18.13^c^	129.23^d^
RDF + *Bacillus xiamenensis* BLW-7	0.422^a^	151.15^bc^	15.69^b^	218.17^ab^	4.49^b^	1.00^c^	1.87^ab^	5.36^ab^	137.80^bc^	101.63^bc^	19.32^bc^	137.41^bc^

RDF: recommended dose of fertilizers: 120 kg N ha^−1^, 60 kg P ha^−1^, and 60 kg K ha^−1^. SOC, soil organic carbon; DHA, dehydrogenase activity; APA, alkaline phosphatase activity; FDA, fluorescein diacetate activity; SMBC, soil microbial biomass carbon; TPF, triphenylformazan; pNP, p-nitrophenol phosphatase; FLR, fluorescein. Data with different letters show significant difference in column data in randomized block design test at *p* < 0.05 under Duncan's multiple range test.

### Effect of microbial inoculation on nutritional content in wheat

Similar to the nethouse experiments, seed inoculation significantly increased the accumulation and content of macronutrients (N, P, and K) and micronutrients (Fe, Zn, Cu, and Mn) in the wheat amended with and without zinc phosphate under field conditions. The results showed that *B. megaterium* CHW-22 was found to be the most potential inoculant followed by *B. licheniformis* MJW-38 and *B. altitudinis* AJW-3, as compared to other inoculants. The results revealed that maximum N, P, and K content in the wheat straw and grain was recorded in the plants treated with RDF + *B. megaterium* CHW-22 without zinc phosphate followed by *B. licheniformis* MJW-38 and *B. altitudinis* AJW-3 as compared to other inoculants and untreated control plants. It was also observed that maximum accumulation of Zn was reported in the grain and straw obtained from the wheat plants bioprimed with *B. megaterium* CHW-22 (49.48 and 37.57 μg g^−1^, respectively) followed by *B. licheniformis* MJW-38 (48.54 and 36.84 μg g^−1^, respectively) and *B. altitudinis* AJW-3 (46.68 and 35.52 μg g^−1^, respectively) as compared to other treatments under field conditions. Similarly, maximum Fe, Cu, and Mn were reported in the grain and straw obtained from the wheat plants bioprimed with *B. megaterium* CHW-22 followed by *B. licheniformis* MJW-38 and *B. altitudinis* AJW-3 as compared to other inoculants. However, the least concentration of macronutrients (N, P, and K) and micronutrients including Zn (Zn, Fe, Cu, and Mn) was reported in the untreated absolute control and RDF alone amended plants without zinc phosphate (Table 7). A more or less similar pattern was recorded in the case of zinc phosphate-amended plants inoculated with selected strains of rhizobacterial inoculants (Table 7).

**Table 7:** Effect of zinc-solubilizing plant growth-promoting rhizobacteria on nutrition concentration in wheat under field experiment.

**Treatment**	**N (%)**		**P (%)**		**K (%)**		**Zn (**μ**g g**^**−1**^**)**		**Fe (**μ**g g**^**−1**^**)**		**Cu (**μ**g g**^**−1**^**)**		**Mn (**μ**g g**^**−1**^**)**	
	**Grain**	**Straw**	**Grain**	**Straw**	**Grain**	**Straw**	**Grain**	**Straw**	**Grain**	**Straw**	**Grain**	**Straw**	**Grain**	**Straw**
**Without Zn**														
Control (N_0_P_0_K_0_)	1.65^d^	0.43^c^	0.29^e^	0.106^a^	0.41^d^	1.43^d^	32.56^e^	24.96^e^	91.58^e^	314.66^e^	10.05^e^	7.34^d^	23.47^e^	32.82^d^
RDF (N_120_P_60_K_60_)	1.74^cd^	0.47^bc^	0.31^d^	0.112^c^	0.45^c^	1.50^c^	34.32^d^	26.12^d^	95.78^d^	327.40^d^	10.29^d^	7.56^c^	23.74^d^	33.28^c^
RDF + *Bacillus altitudinis* AJW-3	1.82^b^	0.52^ab^	0.36^ab^	0.118^ab^	0.50^a^	1.56^ab^	46.68^b^	35.52^b^	105.88^b^	353.80^b^	10.55^ab^	7.70^ab^	24.52^ab^	34.27^ab^
RDF + *Bacillus subtilis* ABW-30	1.77^c^	0.51^ab^	0.34^bc^	0.117^b^	0.49^ab^	1.54^b^	43.82^c^	33.34^c^	101.62^c^	348.30^bc^	10.42^bc^	7.61^b^	24.18^bc^	33.89^b^
RDF + *Bacillus megaterium* CHW-22	1.89^a^	0.55^a^	0.38^a^	0.122^a^	0.51^a^	1.59^a^	49.48^a^	37.57^a^	111.04^a^	368.60^a^	10.68^a^	7.81^a^	24.82^a^	34.68^a^
RDF + *Bacillus licheniformis* MJW-38	1.86^ab^	0.54^a^	0.37^a^	0.120^a^	0.50^a^	1.57^ab^	48.54^ab^	36.84^ab^	109.43^ab^	359.40^ab^	10.65^a^	7.77^a^	24.78^a^	34.51^a^
RDF + *Brevibacillus borstelensis* CHW-2	1.76^c^	0.49^b^	0.33^c^	0.115^bc^	0.48^b^	1.53^b^	42.13^cd^	32.06^cd^	101.23^cd^	344.43^c^	10.38^c^	7.63^b^	23.88^c^	33.54^bc^
RDF + *Bacillus xiamenensis* BLW-7	1.79^bc^	0.51^ab^	0.35^b^	0.117^ab^	0.50^a^	1.56^ab^	45.66^bc^	34.67^bc^	103.38^bc^	351.60^b^	10.48^b^	7.68^ab^	24.36^b^	34.12^ab^
**Zn Applied**														
Control (N_0_P_0_K_0_)	1.70^d^	0.45^d^	0.30^e^	0.110^e^	0.42^d^	1.50^e^	34.84^e^	27.14^e^	96.16^e^	331.97^d^	10.50^f^	7.60^e^	24.53^e^	34.66^e^
RDF (N_120_P_60_K_60_)	1.80^c^	0.49^c^	0.32^d^	0.116^d^	0.47^c^	1.57^d^	37.95^d^	29.64^d^	100.76^d^	343.12^c^	10.79^e^	7.85^d^	24.74^d^	35.08^d^
RDF + *Bacillus altitudinis* AJW-3	1.88^ab^	0.55^ab^	0.38^ab^	0.123^ab^	0.53^a^	1.63^b^	51.81^b^	40.44^b^	112.02^b^	372.55^b^	11.13^b^	8.00^b^	25.65^ab^	36.19^ab^
RDF + *Bacillus subtilis* ABW-30	1.83^b^	0.53^b^	0.36^bc^	0.121^b^	0.51^ab^	1.61^bc^	48.42^c^	37.84^c^	110.62^c^	366.76^bc^	10.93^d^	7.90^c^	25.32^b^	35.75^bc^
RDF + *Bacillus megaterium* CHW-22	1.97^a^	0.58^a^	0.40^a^	0.127a	0.54^a^	1.68^a^	54.43^a^	42.48^a^	116.37^a^	387.77^a^	11.28^a^	8.16^a^	26.04^a^	36.66^a^
RDF + *Bacillus licheniformis* MJW-38	1.95^a^	0.57^a^	0.38^ab^	0.123^ab^	0.53^a^	1.65^a^	53.39^ab^	41.68^ab^	114.79^a^	378.81^ab^	11.26^a^	8.09^ab^	25.99^a^	36.51^a^
RDF + *Brevibacillus borstelensis* CHW-2	1.82^bc^	0.51^bc^	0.34^c^	0.119^c^	0.50^b^	1.60^c^	46.55^cd^	36.37^cd^	109.90^c^	366.13^bc^	10.93^d^	7.87^cd^	24.93^c^	35.38^c^
RDF + *Bacillus xiamenensis* BLW-7	1.86^ab^	0.53^b^	0.37^b^	0.121^b^	0.52^ab^	1.63^b^	50.13^bc^	39.14^bc^	110.76^c^	371.44^b^	11.02^c^	7.95^bc^	25.38^b^	35.93^b^

RDF: Recommended dose of fertilizers: 120 kg N ha^−1^, 60 kg P ha^−1^, and 60 kg K ha^−1^. Data with different letters show significant difference in column data in randomized block design test at *p* < 0.05 under Duncan's multiple range test.

### Effect of microbial inoculation on NUE of wheat

NUE is an important aspect of field experiments. It was clearly observed that microbial inoculation significantly influenced the NUE of wheat in general. In the present investigation, partial factor productivity, agronomic efficiency, apparent nutrient recovery, and physiological efficiency were calculated and correlated with the microbial inoculation and zinc applied under field conditions. The results revealed that apparently higher partial factor productivity for N (38.68), P (77.37), K (77.37), and Zn (928.40) was reported in the plants bioprimed with *B. megaterium* CHW-22 followed by *B. licheniformis* MJW-38 (N: 38.53, P: 77.07, K: 77.07 and Zn: 924.80) and *B. altitudinis* AJW-3 (N: 38.48, P: 76.97, K: 76.97, and Zn: 923.60) as compared to other inoculants without zinc phosphate (Table 8). The partial factor productivity for N, P, K, and Zn was slightly higher in all the treatments amended with zinc phosphate under field conditions. Moreover, the pattern was more or less similar as recorded in the treatments without zinc phosphate (Table 8). Similarly, maximum agronomic efficiency for N, P, and K was also reported in the plants bioprimed with *B. megaterium* CHW-22 (1.37, 2.73, and 2.73, respectively) followed by *B. licheniformis* MJW-38 (1.22, 2.43, and 2.43, respectively) and *B. altitudinis* AJW-3 (1.17, 2.33, and 2.33, respectively) as compared to other inoculants without zinc phosphate (Table 8). Similarly, agronomic efficiency for N, P, K, and Zn was also calculated in the plants inoculated with selected strains of rhizobacteria and amended with zinc phosphate. These values were slightly higher in the case of zinc phosphate-amended plants, while the trend was more or less similar (Table 8). Table 8 clearly showed that maximum apparent nutrient recovery and physiological efficiency were observed in the plants bioprimed with *B. megaterium* CHW-22 followed by *B. licheniformis* MJW-38 and *B. altitudinis* AJW-3 with and without zinc phosphate as compared to other inoculants under field conditions. The significant increase in the partial factor productivity, agronomic efficiency, apparent nutrient recovery, and physiological efficiency of the inoculated plants showed the importance of rhizobacterial inoculants across the treatments. These results indicate the efficacy of the selected zinc-solubilizing rhizobacteria in enhancing the bioavailability of zinc and mobilizing it toward wheat grains.

**Table 8:** Effect of zinc-solubilizing plant growth-promoting rhizobacteria on nutrient use efficiency (NUE) of wheat.

**Treatment**	**Partial Factor Productivity (PEP)**				**Agronomic Efficiency (AE)**				**Apparent Nutrient Recovery (ANR)**				**Physiological Efficiency (PE)**			
	**(N)**	**(P)**	**(K)**	**(Zn)**	**(N)**	**(P)**	**(K)**	**(Zn)**	**(N)**	**(P)**	**(K)**	**(Zn)**	**(N)**	**(P)**	**(K)**	**(Zn)**
**Without Zn**																
Control (N0P0K0)	29.06	58.12	58.12	697.40	-	-	-	-	-	-	-	-	-	-	-	-
RDF (N120P60K60)	37.32	74.63	74.63	895.60	-	-	-	-	-	-	-	-	-	-	-	-
RDF + *Bacillus altitudinis* AJW-3	38.48	76.97	76.97	923.60	1.17	2.33	2.33	-	0.09	0.06	0.15	-	13.69	42.26	15.26	-
RDF + *Bacillus subtilis* ABW-30	38.28	76.57	76.57	918.80	0.97	1.93	1.93	-	0.06	0.04	0.11	-	17.33	52.41	16.86	-
RDF + *Bacillus megaterium* CHW-22	38.68	77.37	77.37	928.40	1.37	2.73	2.73	-	0.13	0.08	0.20	-	10.24	35.57	13.75	-
RDF + *Bacillus licheniformis* MJW-38	38.53	77.07	77.07	924.80	1.22	2.43	2.43	-	0.11	0.06	0.16	-	11.38	37.86	14.95	-
RDF + *Brevibacillus borstelensis* CHW-2	38.10	76.20	76.20	914.40	0.78	1.57	1.57	-	0.04	0.03	0.09	-	21.34	61.55	17.40	-
RDF + *Bacillus xiamenensis* BLW-7	38.40	76.80	76.80	921.60	1.08	2.17	2.17	-	0.07	0.05	0.15	-	16.37	47.44	14.40	-
**Zn Applied**																
Control (N0P0K0)	30.11	60.22	60.22	722.60	-	-	-	-	-	-	-	-	-	-	-	-
RDF (N120P60K60)	38.73	77.47	77.47	929.60	-	-	-	-	-	-	-	-	-	-	-	-
RDF + *Bacillus altitudinis* AJW-3	40.25	80.50	80.50	966.00	1.52	3.03	3.03	36.40	0.10	0.07	0.18	0.04	14.92	43.63	17.06	856.80
RDF + *Bacillus subtilis* ABW-30	39.89	79.78	79.78	957.40	1.16	2.32	2.32	27.80	0.06	0.05	0.13	0.03	18.64	47.12	18.11	865.91
RDF + *Bacillus megaterium* CHW-22	40.66	81.32	81.32	975.80	1.93	3.85	3.85	46.20	0.16	0.09	0.25	0.05	11.80	41.20	15.58	910.49
RDF + *Bacillus licheniformis* MJW-38	40.43	80.85	80.85	970.20	1.69	3.38	3.38	40.60	0.13	0.07	0.20	0.05	12.57	47.90	16.91	859.42
RDF + *Brevibacillus borstelensis* CHW-2	39.58	79.17	79.17	950.00	0.85	1.70	1.70	20.40	0.04	0.03	0.10	0.03	21.32	62.64	17.21	782.77
RDF + *Bacillus xiamenensis* BLW-7	40.09	80.18	80.18	962.20	1.36	2.72	2.72	32.60	0.08	0.06	0.17	0.04	17.28	47.00	16.37	872.39

### Correlation and principal component analyses

**Figure 2:**
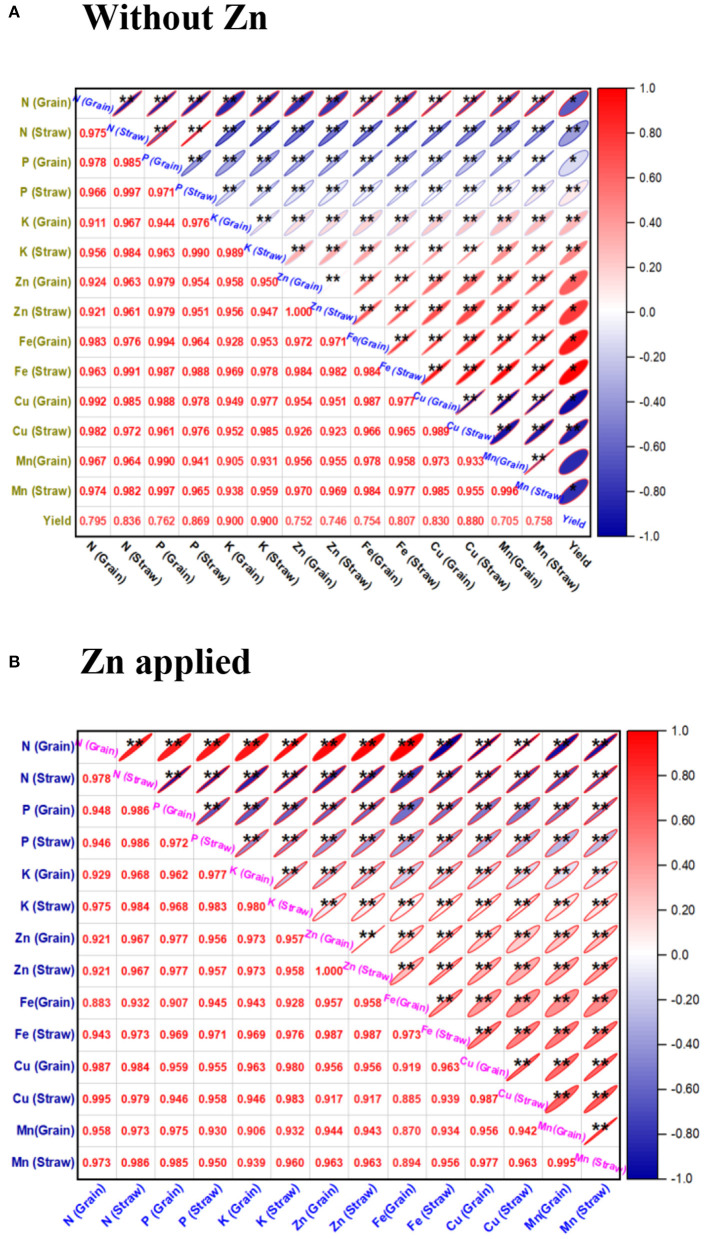
Correlation coefficient (r)* matrix showing the effect of rhizobacterial inoculants on nutrient content in the straw and grain of the wheat plants grown without zinc phosphate **(A)** and with zinc phosphate **(B)** and their interaction under field conditions. The correlation coefficient (r) values are significantly positive at *p* < 0.01, and they are indicated with ** in the matrix.

The univariate Pearson's correlation coefficient (r) matrix (Figure 2) indicated that nutrient content in the grain and straw of the wheat obtained from microbial inoculated plants without zinc phosphate (Figure 2A) as well as soil amended with zinc phosphate (Figure 2B) were found to be significant and positively correlated among them as compared to absolute control under field conditions.

**Figure 3:**
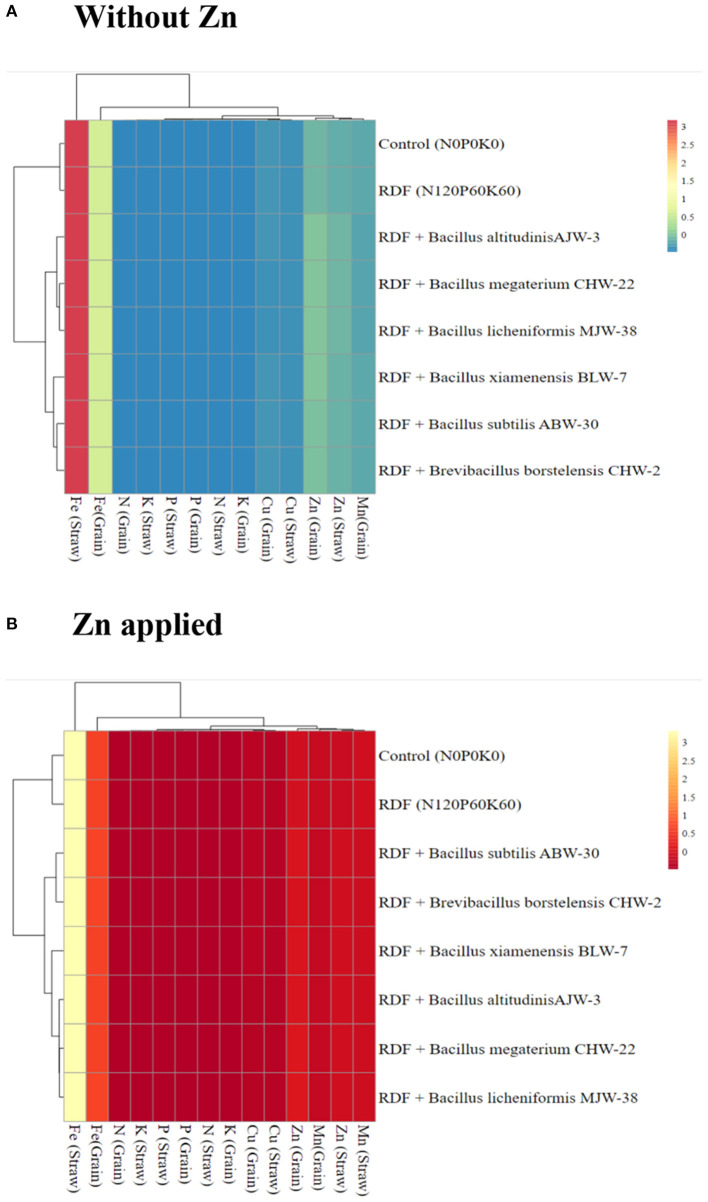
The heatmap depicting the interaction of rhizobacterial inoculants and nutrient content in the straw and grains of the wheat plants grown without zinc phosphate **(A)** and with zinc phosphate **(B)** and their interaction under field conditions. *Correlation coefficient (r) values correspond directly to the color code from (lowest to highest) blue to yellow and red, respectively.

The generated heatmap (Figure 3) displayed the nutrient content vertically and treatments horizontally wherein different clusters were formed based on similarities. A positive interaction was observed with respect to the level of nutrients accumulated in the wheat grain and straw obtained from microbial inoculated plants as compared to absolute control without zinc phosphate (Figure 3A) as well as soil amended with zinc phosphate (Figure 3B) under field conditions.

**Figure 4:**
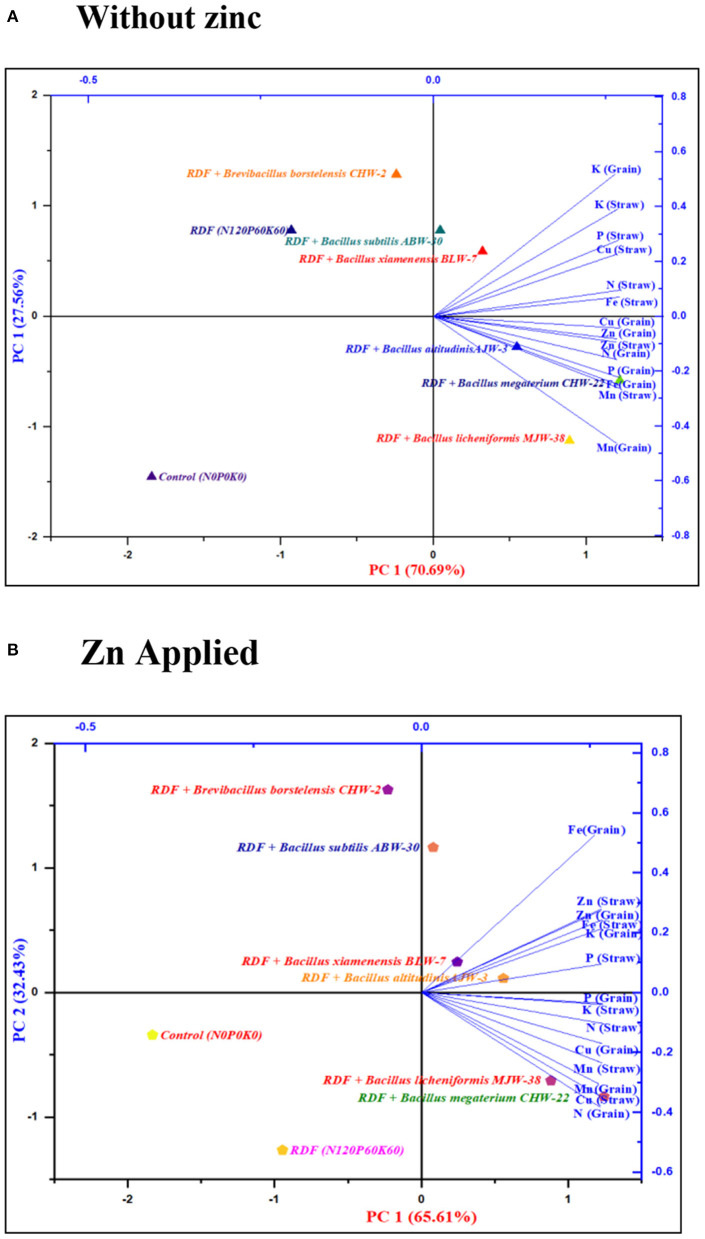
The two-dimensional graphical biplot showing the grouping of variables of wheat crops on principal component scores (PC1 and PC2) derived from variables (nutrient content in grain and straw along with bacterial treatments) of wheat crop grown without zinc phosphate **(A)** and with zinc phosphate **(B)** under field conditions.

The results of the principal component analysis indicated that the position of different macronutrients (NPK) and micronutrients (Zn, Fe, Cu, and Mn) contents in straw and grains of wheat influenced by rhizobacterial strains with or without zinc application are depicted in the four zones of biplot of PCA (Figure 4, Supplementary Table 5). In the absence of zinc application, the PCA comprising two principal components (PC1 70.69% and PC2 27.56%) accounted for 98.25% of the variance (Figure 4A). The N, P, K, and Fe content in straw and K content in grains were influenced by RDF + *B. subtilis* ABW-30 and RDF + *B. xiamenensis* BLW-7 as depicted in right upper side biplot having large positive loading for both PC1 and PC2, indicating that these two strains ABW-30 and BLW-7 have directly impacted the accumulation of nutrients in straw. In contrast, the N, P, Fe, Cu, Zn, and Mn content in grain and the Zn and Mn content in straw were influenced by RDF + *B. megaterium* CHW-22, RDF + *B. altitudinis* AJW-3 and RDF + *B. licheniformis* MJW-38 as depicted in the right lower side of biplot having positive loading PC 1 and negative loading for PC 2, indicating that strains CHW-22, AJW-3, and MJW-38 have influenced nutrient contents in grains.

In presence of zinc application, the PCA comprising two principal components (PC1 65.61% and PC2 32.43%) accounted for 98.04% of the variance (Figure 4B). The P, Zn, and Fe in straw and K, Zn, and Fe in grains were influenced by RDF + *B. altitudinis* AJW-3 and RDF + *B. xiamenensis* BLW-7 as depicted in the right upper side biplot having large positive loading for both PC1 and PC2. However, N, K, Mn, and Cu in straw and N, P, Mn, and Cu in grains were influenced by RDF + *B. megaterium* CHW-22 and RDF + *B. licheniformis* MJW-38 as depicted in the right lower side biplot having large positive loading for PC1 and negative loading for PC2. It was revealed from the above mentioned results that bacterial strains have differential behavior in the partitioning of nutrients in straw and grains in the presence and absence of zinc phosphate in conjunction with RDF. In the absence of zinc application, some bacteria partitioned nutrients in straw, whereas some of them were transferred to the grains, but in the presence of zinc application, the behavior of strains was found mixed. The possible reasons for such behavior need further investigation.

## Discussion

Zinc availability in soil is decreasing day by day with the increasing salinization and land degradation over a period of time. Micronutrient deficiencies including Zn are often referred to as “hidden hunger” for plants as well as human beings (Cakmak, 2008; White and Broadley, 2011). It is very difficult to detect deficiencies over a short period of time. Zn is not only essential for plant growth and development but also plays a key role in the physico-biochemical process in plants. It provides the major dietary source of Zn for a large segment of the world population. The long-term deficiencies can have irreversible serious consequences for plant health (White and Broadley, 2011; Singh et al., 2021b; Yadav et al., 2022). It is therefore difficult to eliminate Zn deficiency through conventional agriculture practices including crop diversification and nutritional supplements through inorganic fertilizers (Ramesh et al., 2014; Khande et al., 2017; Singh et al., 2021b). Conversely, increasing intrinsic Zn content in food crops, especially wheat, by breeding and microbial intervention is well documented as an environment-friendly, cost-effective, and sustainable strategy to tackle Zn deficiency (Cakmak, 2008; White and Broadley, 2011). Furthermore, a large amount of Zn is present in the soil in insoluble forms being unavailable to plants. There is an urgent need to search for potential Zn-solubilizing microorganisms, which can solubilize insoluble Zn pool present in the soil and make it available to the plants without harming the environment (Sharma et al., 2010; Ramesh et al., 2014; Singh et al., 2021b). Thus, it plays as a bridge between the soil and plant roots. Among soil-dwelling microorganisms, harnessing the potential of native plant growth-promoting rhizobacteria (PGPR) including Zn-solubilizing rhizobacteria is an alternate strategy for enhancing Zn solubilization, uptake and translocation, and biofortification of Zn in plants including wheat (Sharma et al., 2011; Ramesh et al., 2014; Singh et al., 2021b). In general, PGPR have the ability to solubilize and mobilize unavailable zinc and increase the assimilation of zinc in grains. PGPR are an integral part of any ecological niche. They play a crucial role in nutrient geo-cycling including nutrient mineralization in the environment (Sharma et al., 2010; Rana et al., 2012; Ramesh et al., 2014). Zinc-solubilizing rhizobacteria secret a large amount of organic acids, protein extrusion, and chelating agents that enhance the overall availability of Zn in the rhizosphere ecosystem (Nahas, 1996; Seshadri et al., 2000), which can be substantially taken up by plants. Therefore, the key aim of the present study was to characterize the native potential zinc-solubilizing rhizobacterial strains and their application for overall plant growth with special reference to zinc biofertilization in wheat in conjunction with soil-applied fertilizers and zinc phosphate under pot and field conditions.

In the present investigation, a total of 175 different rhizobacterial strains were isolated from the wheat rhizospheric soils collected from different parts of the Indo-Gangetic plains of Northern India (Supplementary Table 1). The soils of the Indo-Gangetic plains of the Indian sub-continent are considered one of the mineral-rich soils in India. It harbors a rich microbial gene pool, which plays an important role in nutrient geo-cycling, nutrient mineralization, waste decomposition, and soil processes, and provides protection from several biotic and abiotic stresses including nutritional deficiencies (Malviya et al., 2011; Srivastava et al., 2014; Sharma et al., 2015; Kumari et al., 2016; Choudhary et al., 2018a,b; Singh et al., 2020). Of the 175 bacterial strains, 42 strains were found to solubilize either zinc carbonate (ZnCO_3_), zinc oxide (ZnO), and zinc phosphate {Zn_3_(PO_4_)_2_} or any two or all three compounds of insoluble Zn. These strains were found to produce organic acids such as gluconic acid, oxalic acid, citric acid, malic acid, lactic acid, and succinic acid (data not shown). The production of organic acids in the broth supplemented with ZnCO_3_, ZnO, and Zn_3_(PO_4_)_2_ and inoculated with bacterial strains indicated that ‘organic acids' are essential for Zn solubilization as reported by earlier studies (Desai et al., 2012; Ramesh et al., 2014; Kumari et al., 2016; Yadav R. C. et al., 2021). However, gluconic acid is the key organic acid produced by the majority of Zn-solubilizing bacterial strains for the solubilization of insoluble minerals besides other organic acids. These results are in line with the observations made by many researchers (Zhao et al., 2011; Rashid et al., 2012; Yadav et al., 2022). These 42 strains were further identified on the basis of 16S rRNA gene sequences. The results of the present investigation clearly indicated that the rhizosphere of wheat grown in the IGP region harbor rich diversity of rhizobacteria. It was reported that 19 different species of rhizobacteria were isolated and characterized from the wheat rhizosphere. *Bacillus* and *Bacillus*-derived genera are ubiquitous in nature and possess multiple growth-promoting traits (Kohler et al., 2007; Ramirez et al., 2010; Zhao et al., 2011).

In general, bacilli are root-associated mutualistic plant symbionts widely present in the rhizosphere soil (Singh et al., 2016, 2021a). They have the vast capability to colonize plant roots, nourish the host, and protect plants from biotic and abiotic stresses (Singh et al., 2016, 2021a,b; Yadav et al., 2022). The variation in Zn solubilization may be found due to the type and amount of organic acid produced, culture conditions, pH of culture medium, nature of microbes, and gene induced (responsible for Zn mineralization) in response to the nature of Zn compounds used as the sole source of Zn (Ramesh et al., 2014; Khande et al., 2017). Based on the halo zone produced on plates containing tris-minimal media supplemented with 0.1% ZnCO_3_, ZnO, and Zn_3_(PO_4_)_2_, six potential zinc-solubilizing bacterial strains, i.e., *B. altitudinis* AJW-3, *B. subtilis* ABW-30, *B. megaterium* CHW-2, *B. licheniformis* MJW-22, *B. borstelensis* CHW-38, and *B. xiamenensis* BLW-7, were selected for further *in vitro* and *in planta* assay. In the present investigation, the selected strains showed varying degrees of ZSE, amount of solubilized Zn, pH of culture medium, and gluconic acid produced. The organic acid specifically gluconic acid of microbial origin possibly works in a non-specific way during the Zn solubilization process and thereby influencing the bioavailability of zinc (Agusto da Costa and Duta, 2001). *In vitro* plate assay clearly showed that the selected strains *B. altitudinis* AJW-3, *B. subtilis* ABW-30, *B. megaterium* CHW-2, *B. licheniformis* MJW-22, *B. borstelensis* CHW-38, *and B. xiamenensis* BLW-7 possess several other PGP traits such as P and K solubilization, IAA, siderophore, HCN, and ammonia production, thereby promoting plant growth and development directly and/or indirectly. These findings are in accordance with the observation of several other studies (Ramesh et al., 2014; Mumtaz et al., 2017; Dinesh et al., 2018; Singh et al., 2021a).

The effects of seed inoculation were studied in the wheat plants under the pot as well as field conditions. Seed biopriming with these strains significantly increases plant height, dry matter accumulation, yield and yield attributing characters (ET Plant^−1^, spike length, spikelet spike^−1^, grain spike^−1^, and test weight), grain yield, straw yield, biological yield, and harvest index in the wheat supplemented with Zn and without Zn under nethouse conditions. The values are significantly higher in the plants supplemented with Zn and with the recommended dose of fertilizers as compared to the plants supplemented with the recommended dose of fertilizers and without Zn. When compared with the absolute control (N_0_P_0_K_0_ and no inoculation), bacterial inoculation has a significant impact on plant growth parameters and yield-attributing traits in the plants supplemented with and without Zn. The application of PGPR under nethouse experiments significantly increases plant growth and development (Lwin et al., 2012; Ram et al., 2013; Porcel et al., 2014; Singh et al., 2021b; Yadav et al., 2022). These inoculants produced several growth hormones (Dodd et al., 2010; Jha and Saraf, 2011; Kang et al., 2012; Maheshwari et al., 2015; Tsukanova et al., 2017; Khan et al., 2020) and improved chlorophyll content (Heidari and Golpayegani, 2012; Vafadar et al., 2014; Mathivanan et al., 2017), induction of physiological processes (Meena et al., 2020; Singh et al., 2021a,b), mineralization, and solubilization of mineral nutrient (Saravanan et al., 2007; Desai et al., 2012; Lucas et al., 2014; Wang et al., 2014; Kumari et al., 2016).

Application of microbial inoculants significantly increased the availability of macro (N, P, and K) and micro (Fe, Zn, Cu, and Mn) nutrients in the wheat rhizosphere soil (Singh et al., 2021b; Tirry et al., 2021). PGPR are known to break down the complex organic and inorganic compounds such as proteins, lignin, cellulose, hemicellulose, and lipids in the natural ecosystem. They mineralize/solubilize the mineral nutrients in the soil ecosystem and transform them into available states (Ren et al., 2021; Shabaan et al., 2022). These strains were previously known for their ability of P, K, and Zn solubilization, IAA production, and ACC deaminase activity, thereby enhancing P-uptake and plant biomass in many crops (Saravanan et al., 2004; Lucas et al., 2014; Pereira and Castro, 2014; Wang et al., 2014). Furthermore, inoculation of bacterial inoculants significantly also increased (1.25-1.67 fold) the activity of DHA, APA, and FDA in the rhizosphere soil along with SMBC under nethouse experiments with and without Zn. These results are in agreement with the other researchers (Kohler et al., 2007; Rana et al., 2012; Song et al., 2015; Arif et al., 2016; Islam et al., 2016; Sood et al., 2018; Maddhesiya et al., 2021; Ren et al., 2021; Shabaan et al., 2022).

Seed biopriming significantly enhanced the zinc content in grain and straw as compared to non-inoculated absolute control and plants supplemented with recommended doses of fertilizers and Zn. The amount of Zn accumulated in the wheat grain and straw of the plants inoculated with bacterial inoculants and applied with Zn was significantly higher as compared to plants without Zn application. Among the different bacterial inoculants, maximum Zn was recorded in the grain and straw of the plants inoculated with *B. megaterium* CHW-22 as compared to other inoculants. These results are in line with the previous reports wherein inoculation of plants with PGPR led to enhanced Zn content in wheat grain (Islam et al., 2014, 2016; Ramesh et al., 2014; Sirohi et al., 2015; Kamran et al., 2017; Singh et al., 2021b; Azmat et al., 2022; Yadav et al., 2022). The ZIP (Zn-regulated, iron-regulated transporter-like protein) transporter families are well-studied transporters in terrestrial plants. It is an important transporter present in the different cell organelles and regulates the uptake, transport, and accumulation of Fe and Zn in the plant's system (Singh et al., 2021b; Aloo et al., 2022; Yadav et al., 2022). Along with the uptake and translocation of Fe and Zn in the plants, they also play a key role in several other developmental processes, such as plant growth, uptake and translocation of other key microelements, tissues differentiation, and biofortification in the plant system (Velmourougane et al., 2017; Yadav et al., 2022). The ZIP protein family has been widely studied in *Arabidopsis thaliana*, a model plant. These transporters have also been mapped in several other plant species such as *Oryza sativa, Lycopersicon esculentum* Mill., and *Zea mays* in the past few years. However, the importance of the ZIP family in wheat is not well-studied at present and it needs in-depth investigation. Furthermore, it is the need of the hour to get the ZIP transporters mapped in wheat in relation to the microbial inoculants interaction which is in the infancy stage (Singh et al., 2021a,b; Yadav et al., 2022). Besides the increase in the Zn content in the plant, they also increase the uptake and content of other mineral nutrients (N, P, K, Fe, Cu, and Mn) in the grain and straw of the wheat plants bioprimed with selected strains of *B. altitudinis* AJW-3, *B. subtilis* ABW-30, *B. megaterium* CHW-2, *B. licheniformis* MJW-22, *B. borstelensis* CHW-38, and *B. xiamenensis* BLW-7. Several studies reported increased mobilization of macro- and micronutrients by PGPR including zinc-solubilizing bacilli in many crops including wheat (Sharma et al., 2011; Rana et al., 2012; Kumar et al., 2014, 2021). It was clearly indicated that bacterial inoculation significantly affects the NUE of wheat in general. Inoculation of *B. megaterium* CHW-2 significantly increased the partial factor productivity, agronomic efficiency, apparent nutrient recovery, and physiological efficiency as compared to other inoculants under field conditions. These results are in agreement with the finding of several other studies which reported that PGPRs significantly increased the uptake and translocation of mineral nutrients from the soil and biofortified wheat and other crops (Adesemoye et al., 2008; Shaharoona et al., 2008; Arif et al., 2017; Çakmakçi et al., 2017; Di Benedetto et al., 2017; Pereira et al., 2020; Singh et al., 2020). Based on the results of the present investigation and the observations of the previous researchers, it can be summarized that zinc-solubilizing rhizobacteria can be used as an alternate strategy for *in vivo* Zn solubilization and enhance Zn uptake and translocation in the plants to cope with the Zn deficiency in wheat. In this way, these potential rhizobacteria mobilize unavailable zinc present in the soil and increase the assimilation of zinc, accelerating plant growth and enhancing the overall yield of plants. Furthermore, they play a crucial role in the environmental geo-cycling of mineral nutrients, which can be suitably taken up by plants in a sustainable way. Moreover, Zn solubilization by native PGPR is a comparatively new approach and not many strains have yet been characterized and reported so far. In the present investigation, a number of native potential zinc-solubilizing rhizobacterial isolates have been explored and characterized, which can be used as biofertilizers to overcome zinc deficiency in wheat crops.

## Conclusion

Inoculation of PGPR linked with zinc-solubilizing ability effectively improved soil biological properties, growth, yield, and micronutrient-enriched seeds of wheat. Of the 175 rhizobacterial isolates recovered and screened, only 6 bacteria were found effective in the solubilization of insoluble zinc compounds. Of the six zinc-solubilizing bacteria, inoculation of *B. megaterium* CHW-22, *B. licheniformis* MJW-38, and *B. altitudinis* AJW-3 in conjunction with RDF and zinc phosphate enhanced wheat growth and grain yield and grain with zinc in the wheat crop in degraded soil with high pH of Eastern Uttar Pradesh of India. The results revealed that bacterial inoculation enhanced the NUE of the applied fertilizers and zinc phosphate in comparison with RDF alone. Such effective bacterial inoculants are beneficial for sustainable agriculture as they have the ability to combat abiotic stresses and facilitate functioning in degraded soil for ensuring food and nutritional security and, in turn, achieve the objectives of Sustainable Development Goals by the year 2030.

## Data availability statement

The datasets presented in this study can be found in online repositories. The names of the repository/repositories and accession number(s) can be found in the article/Supplementary material.

## Author contributions

SS, AV, and RY conceptualized the idea for this research work and corrected the first draft of the manuscript. SS and RY designed the experiments. RY and US conducted experiments and developed the first draft of the manuscript. AK and IB performed the statistical analyses using different software and interpreted the data thereafter. SS, PS, and JR edited the final draft of the manuscript. The final draft of the manuscript was read by all the authors and all of them gave their consent for publication. All authors contributed to the article and approved the submitted version.
