# Supplementary material for: Zinc-solubilizing Bacillus spp. in conjunction with chemical fertilizers enhance growth, yield, nutrient content, and zinc biofortification in wheat crop
Source: Front Microbiol. 2023 Jul 4;14:1210938. doi: 10.3389/fmicb.2023.1210938 (PMC10352851; doi:10.3389/fmicb.2023.1210938)
Supplement: Supplementary file 1 [file Table_1.pdf]

Supplementary Table 1. Geographical locations from where rhizobacterial strains were isolated.

S.No.	Isolate	Locations	Coordinates	Year of isolation
**1.**	CHW-12	Barhani, Chandauli, Uttar Pradesh (India)	25° 17' 29.976" N 83° 24' 25.74" E	**2017**
**2.**	ABW-30	Baskhari, Ambedkar Nagar, Uttar Pradesh (India)	26° 27' 8.586" N 82° 46' 50.0016" E	**2018**
**3.**	CHW-21	Chakia, Chandauli, Uttar Pradesh (India)	25° 2' 48.84" N 83° 13' 3.7092" E	**2017**
**4.**	JNW-2	Badalapur, Jaunpur,Uttar Pradesh (India)	25° 53' 34.908" N 82° 27' 10.62" E	**2018**
**5.**	VAW-19	Raja Talab,Varanasi, Uttar Pradesh (India)	27° 6' 0.756" N 80° 53' 20.04" E	**2018**
**6.**	ABW-43	Ramnagar, Ambedkar Nagar, Uttar Pradesh (India)	28° 39' 41.616" N 77° 26' 9.3156" E	**2018**
**7.**	ABW-46	Ramnagar, Ambedkar Nagar, Uttar Pradesh (India)	28° 39' 41.616" N 77° 26' 9.3156" E	**2018**
**8.**	CHW-4	Barhani, Chandauli, Uttar Pradesh (India)	25° 17' 29.976" N 83° 24' 25.74" E	**2017**
**9.**	JNW-23	Sivatha,Jaunpur,Uttar Pradesh (India)	25° 45' 17.64" N 82° 41' 13.416" E	**2018**
**10.**	BLW-7	Kalikthan, Balrampur Uttar Pradesh (India)	27° 25' 3.36" N 82° 10' 10.56" E	**2017**
**11.**	ABW-17	Akabarpur, Ambedkar Nagar, Uttar Pradesh (India)	26° 26' 33.864" N 82° 40' 51.132" E	**2018**
**12.**	SNW-27	Chopan, Sonbhadra, Uttar Pradesh (India)	24° 30' 42.12" N 83° 1' 29.28" E	**2018**
**13.**	CHW-16	Chakia, Chandauli, Uttar Pradesh (India)	25° 2' 48.84" N 83° 13' 3.7092" E	**2017**
**14.**	CHW-2	Barhani, Chandauli, Uttar Pradesh (India)	25° 17' 29.976" N 83° 24' 25.74" E	**2017**
**15.**	MJW-46	Khamhhariya, Mirzapur ,Uttar Pradesh (India)	25° 13' 33.96" N 82° 39' 34.92" E	**2018**
**16.**	ABW-59	Tanda, Ambedkar Nagar, Uttar Pradesh (India)	26° 32' 35.88" N 82° 39' 52.2" E	**2018**
**17.**	CHW-19	Chakia, Chandauli, Uttar Pradesh (India)	25° 2' 48.84" N 83° 13' 3.7092" E	**2017**
**18.**	MJW-48	Khamhhariya, Mirzapur ,Uttar Pradesh (India)	25° 13' 33.96" N 82° 39' 34.92" E	**2018**
19.	MJW-43	Khamhhariya, Mirzapur ,Uttar Pradesh (India)	25° 13' 33.96" N82° 39' 34.92" E	**2018**
**20.**	ABW-54	Tanda, Ambedkar Nagar, Uttar Pradesh (India)	26° 32' 35.88" N 82° 39' 52.2" E	**2018**
**21.**	CHW-25	Kathawal, Sakaldeeha, Chandauli, Uttar Pradesh (India)	25° 15' 39.6" N 83° 15' 35.64" E	**2017**
**22.**	SNW-26	Chopan, Sonbhadra, Uttar Pradesh (India)	24° 30' 42.12" N 83° 1' 29.28" E	**2018**

**23.**	ABW-58	Tanda, Ambedkar Nagar, Uttar Pradesh (India)	26° 32' 35.88" N 82° 39' 52.2" E	**2018**
**24.**	CHW-15	Chakia, Chandauli, Uttar Pradesh (India)	25° 2' 48.84" N 83° 13' 3.7092" E	**2017**
**25.**	CHW-10	Barhani, Chandauli, Uttar Pradesh (India)	25° 17' 29.976" N 83° 24' 25.74" E	**2017**
**26.**	CHW-22	Kathawal, Sakaldeeha, Chandauli, Uttar Pradesh (India)	25° 15' 39.6" N 83° 15' 35.64" E	**2017**
**27.**	JNW-11	Badalapur, Jaunpur, Uttar Pradesh (India)	25° 52' 58.8" N 82° 27' 5.76" E	**2018**
**28.**	ABW-16	Akabarpur, Ambedkar Nagar, Uttar Pradesh (India)	26° 26' 33.864" N 82° 40' 51.132" E	**2018**
**29.**	ABW-53	Tanda, Ambedkar Nagar, Uttar Pradesh (India)	26° 32' 35.88" N 82° 39' 52.2" E	**2018**
**30.**	ABW-15	Akabarpur, Ambedkar Nagar, Uttar Pradesh (India)	26° 26' 33.864" N 82° 40' 51.132" E	**2018**
**31.**	JNW-1	Badalapur, Jaunpur,Uttar Pradesh (India)	25° 52' 58.8" N 82° 27' 5.76" E	**2018**
**32.**	VAW-3	Raja Talab,Varanasi, Uttar Pradesh (India)	27° 6' 0.756" N 80° 53' 20.04" E	**2017**
**33.**	MJW-38	Tilthi,Kon,Mirzapur ,Uttar Pradesh (India)	25° 7' 17.436" N 82° 33' 48.42" E	**2018**
**34.**	JNW-7	Badalapur, Jaunpur,Uttar Pradesh (India)	25° 52' 58.8" N 82° 27' 5.76" E	**2018**
**35.**	AJW-3	Mahrajganj, Ajamgarh,Uttar Pradesh (India)	26° 26' 26.052" N 82° 32' 45.276" E	**2017**
**36.**	CHW-1	Barhani, Chandauli, Uttar Pradesh (India)	25° 17' 29.976" N 83° 24' 25.74" E	**2017**
**37.**	BLW-47	Tulsipur, Balrampur Uttar Pradesh (India)	27° 31' 28.56" N 82° 24' 56.52" E	**2017**
**38.**	BAW-33	Mahsi,Baharaiech, Uttar Pradesh (India)	27° 38' 17.52" N 81° 22' 2.64" E	**2017**
**39.**	BLW-67	Pachperwa, Balrampur Uttar Pradesh (India)	27° 31' 5.52" N 82° 38' 45.96" E	**2017**
**40.**	SKW-18	Nathan Nagar, SantKabir Nagar Uttar Pradesh (India)	26° 38' 34.08" N 83° 1' 33.96" E	**2017**
**41.**	MHW-25	Paratwal, Maharaj Ganj, Uttar Pradesh (India)	26° 58' 5.88" N 83° 36' 5.76" E	**2017**
**42.**	STW-46	Lambhua, Sultanpur, Uttar Pradesh (India)	26° 8' 25.08" N 82° 13' 37.56" E	**2017**

Supplementary Table 2. Zinc solubilizing ability of bacterial isolates on three zinc complex media

S.No	Isolates	$ZnCO_3$	ZnO	$Zn_3 (PO4)_2$
1	CHW-12	10 mm	12 mm	13 mm
2	**AJW-3**	**18 mm**	**15 mm**	**26 mm**
3	CHW-21	-	12 mm	-
4	JNW-2	-	-	20 mm
5	**ABW-30**	**20 mm**	**22 mm**	**24 mm**
6	ABW-43	-	14 mm	-
7	ABW-46	-	20 mm	14 mm
8	CHW-4	11 mm	16mm	-
9	JNW-23	16 mm	-	18 mm
10	JNW-11	10 mm	15mm	12 mm
11	ABW-17	-	18 mm	-
12	SNW-27	-	-	15 mm
13	CHW-16	-	15 mm	10 mm
14	MJW-46	-	-	15 mm
15	**CHW-2**	**22 mm**	**20 mm**	**30 mm**
16	ABW-59	10 mm	17 mm	-
17	CHW-19	-	10 mm	14 mm
18	MJW-48	-	-	13 mm
19	MJW-43	-	-	18 mm
20	ABW-54	12 mm	21 mm	-
21	CHW-25	-	12 mm	15 mm
22	SNW-26	-	-	17 mm
23	**CHW-22**	**16 mm**	**14 mm**	**28 mm**
24	CHW-15	-	15 mm	12 mm
25	**MJW-38**	**24 mm**	**16 mm**	**26 mm**
26	ABW-58	11 mm	22 mm	-
27	**BLW-7**	**15 mm**	**18 mm**	**24 mm**
28	ABW-16	-	15mm	20 mm
29	ABW-53	-	13mm	13 mm
30	ABW-15	14 mm	18 mm	-
31	JNW-1	-	-	12 mm
32	VAW-3	-	12mm	18 mm
33	CHW-10	13mm	-	17 mm
34	JNW-7	-	15 mm	12 mm
35	VAW-19	-	-	14 mm
36	CHW-1	-	-	13 mm
37	BLW-47	-	-	22 mm
38	BAW-33	-	-	24 mm
39	BLW-67	-	-	20 mm
40	SKW-18	12 mm	-	-
41	MHW-25	-	-	22 mm
42	STW-46	11 mm	12 mm	14 mm

Supplementary Table 3. Bacterial isolates identified by 16S rRNA gene sequencing with their accession numbers

S.No	Strain	Sequence Length	Completeness (%)	E Value	Similarity	Sequence ID	Accession Number	Identified As
1	CHW-12	1487	100	0.0	100	SUB11613253	ON763228	*Brevibacillus agri*
2	ABW-30	1483	100	0.0	100	SUB11613253	ON763229	*Bacillus subtilis*
3	CHW-21	1440	100	0.0	100	SUB11613253	ON763230	*Sphingobacterium kitahiroshimense*
4	JNW-2	1516	100	0.0	100	SUB11613253	ON763231	*Advenella kashmirensis*
5	VAW-19	1511	100	0.0	100	SUB11613253	ON763232	*Bacillus xiamenensis*
6	ABW-43	1554	100	0.0	100	SUB11613253	ON763233	*Bacillus thuringiensis*
7	ABW-46	1534	100	0.0	100	SUB11613253	ON763234	*Alcaligenes faecalis*
8	CHW-4	1508	100	0.0	100	SUB11613253	ON763235	*Bacillus altitudinis*
9	JNW-23	1508	100	0.0	100	SUB11613253	ON763236	*Oceanobacillus caeni*
10	BLW-7	1511	100	0.0	100	SUB11613253	ON763237	*Bacillus xiamenensis*
11	ABW-17	1498	100	0.0	100	SUB11613253	ON763238	*Paenibacillus glucanolyticus*
12	SNW-27	1458	100	0.0	100	SUB11613253	ON763239	*Alcaligenes faecalis*
13	CHW-16	1544	100	0.0	100	SUB11613253	ON763240	*Advenella kashmirensis*
14	CHW-2	1479	100	0.0	100	SUB11613253	ON763241	*Brevibacillus borstelensis*
15	MJW-46	1544	100	0.0	100	SUB11613253	ON763242	*Advenella kashmirensis*
16	ABW-59	1544	100	0.0	100	SUB11613253	ON763243	*Advenella kashmirensis*
17	CHW-19	1491	100	0.0	100	SUB11613253	ON763244	*Bacillus cereus*
18	MJW-48	1544	100	0.0	100	SUB11613253	ON763245	*Advenella kashmirensis*
19	MJW-43	1544	100	0.0	100	SUB11613253	ON763246	*Advenella kashmirensis*
20	ABW-54	1516	100	0.0	100	SUB11613253	ON763247	*Advenella kashmirensis*
21	CHW-25	1496	100	0.0	100	SUB11613253	ON763248	*Brevibacterium aurantiacum*
22	SNW-26	1506	100	0.0	100	SUB11613253	ON763249	*Bacillus cereus*
23	ABW-58	1520	100	0.0	100	SUB11613253	ON763250	*Bacillus wiedmannii*

24	CHW-15	1544	100	0.0	100	SUB11613253	ON763251	*Bacillus cereus*
25	CHW-10	1440	100	0.0	100	SUB11613253	ON763252	*Sphingobacterium kitahiroshimense*
26	CHW-22	1509	100	0.0	100	SUB11613253	ON763253	*Bacillus megaterium* or *Priestia megaterium*
27	JNW-11	1516	100	0.0	100	SUB11613253	ON763254	*Advenella kashmirensis*
28	ABW-16	1506	100	0.0	100	SUB11613253	ON763255	*Bacillus cereus*
29	ABW-53	1516	100	0.0	100	SUB11613253	ON763256	*Advenella kashmirensis*
30	ABW-15	1508	100	0.0	100	SUB11613253	ON763257	*Bacillus paramycoides*
31	JNW-1	1516	100	0.0	100	SUB11613253	ON763258	*Advenella kashmirensis*
32	VAW-3	1479	100	0.0	100	SUB11613253	ON763259	*Brevibacillus borstelensis*
33	MJW-38	1515	100	0.0	100	SUB11613253	ON763260	*Bacillus licheniformis*
34	JNW-7	1524	100	0.0	100	SUB11613253	ON763261	*Brevibacillus borstelensis*
35	AJW-3	1524	100	0.0	100	SUB11613253	ON763262	*Bacillus altitudinis*
36	CHW-1	1490	100	0.0	100	SUB11613253	ON763263	*Sphingobacterium kitahiroshimense*
37	BLW-47	1507	100	0.0	100	SUB11613253	ON763273	*Bacillus tequilensis*
38	BAW-33	1558	100	0.0	100	SUB11613253	ON763274	*Bacillus flexus or Priestia flexa*
39	BLW-67	1438	100	0.0	100	SUB11613253	ON763275	*Bacillus paramycoides*
40	SKW-18	1517	100	0.0	100	SUB11613253	ON763276	*Bacillus cereus*
41	MHW-25	1488	100	0.0	100	SUB11613253	ON763277	*Bacillus tequilensis*
42	STW-46	1514	100	0.0	100	SUB11613253	ON763278	*Bacillus cereus*

Supplementary Table 4: *In vitro* characterization of rhizospheric bacteria on the basis of plant growth promotion attributes exhibited by selected isolates

S.No	Strain	Plant growth promoting traits							Enzyme assay					
		K	P	IAA ug/ml	ACC	Siderophore	HCN	Ammonia	Catalase	Oxidase	Amylase	Protease	Lipase	Cellulase
1	AJW-3	+++	+	7.16	+	+++	+++	-	+	-	+	+	+	++
2	ABW-30	+	++	6.43	+	+++	-	-	+	-	++	-	++	+
3	CHW-2	+++	+++	18.61	+	-	++	++	+	-	+++	+++	-	-
4	MJW-22	+++	+++	10.16	-	-	+	+++	+	-	-	+	+	-
5	CHW-38	+++	+	8.61	-	++	++	+	+	-	-	++	-	++
6	BLW-7	+++	+++	12.70	-	-	-	++	+	-	++	+	+	-

(+)= low activity; (++) =moderate activity; (+++) strong activity; (-) = no activity

Supplementary Table 5. Results of principal component analysis.

Principal components		Without zinc		Zinc applied	
		**PC1**	**PC2**	**PC1**	**PC2**
Initial eigen values	Total	9.90	3.86	9.19	4.54
	% of Variance	70.69	27.56	65.61	32.43
	Cumulative %	70.69	98.25	65.61	98.04
Rotation sums of squared loadings	Total	7.74	6.02	7.80	5.92
	% of Variance	55.28	42.97	55.75	42.30
	Cumulative %	55.28	98.25	55.75	98.04
**Factor loadings**[a]					
**Eigen vectors**[b]		**PC1**	**PC2**	**PC1**	**PC2**
N (Grain)		0.979	0.201	0.991	0.133
N (Straw)		0.991	0.128	0.997	0.058
P (Grain)		0.992	0.119	0.998	0.048
P (Straw)		0.994	0.104	0.999	0.033
K (Grain)		0.992	0.126	0.998	0.056
K (Straw)		0.983	0.186	0.993	0.117
Zn (Grain)		0.187	0.942	0.090	0.954
Zn (Straw)		0.278	0.923	0.141	0.948
Fe(Grain)		0.041	0.998	-0.012	0.998
Fe (Straw)		-0.073	0.993	-0.104	0.989
Cu (Grain)		0.799	0.594	0.818	0.568
Cu (Straw)		0.870	0.488	0.895	0.441
Mn(Grain)		0.488	0.856	0.488	0.854
Mn (Straw)		0.352	0.916	0.325	0.923

Extraction Method: Principal Component Analysis;
Rotation Method: Varimax with Kaiser Normalization
[a]Bold faced factor loadings are considered highly weighted;
[b]Rotation converged in 5 iterations.
